# The political, psychological, and social correlates of cryptocurrency ownership

**DOI:** 10.1371/journal.pone.0305178

**Published:** 2024-07-03

**Authors:** Shane Littrell, Casey Klofstad, Joseph E. Uscinski

**Affiliations:** 1 Munk School of Global Affairs and Public Policy, University of Toronto, Toronto, ON, Canada; 2 Department of Political Science, University of Miami, Coral Gables, Florida, United States of America; Yeditepe University, TURKEY

## Abstract

Cryptocurrency is a digital asset secured by cryptography that has become a popular medium of exchange and investment known for its anonymous transactions, unregulated markets, and volatile prices. Given the popular subculture of traders it has created, and its implications for financial markets and monetary policy, scholars have recently begun to examine the political, psychological, and social characteristics of cryptocurrency investors. A review of the existing literature suggests that cryptocurrency owners may possess higher-than-average levels of nonnormative psychological traits and exhibit a range of non-mainstream political identities. However, this extant literature typically employs small nonrepresentative samples of respondents and examines only a small number of independent variables in each given study. This presents the opportunity for both further testing of previous findings as well as broader exploratory analyses including more expansive descriptive investigations of cryptocurrency owners. To that end, we polled 2,001 American adults in 2022 to examine the associations between cryptocurrency ownership and individual level political, psychological, and social characteristics. Analyses revealed that 30% of our sample have owned some form of cryptocurrency and that these individuals exhibit a diversity of political allegiances and identities. We also found that crypto ownership was associated with belief in conspiracy theories, “dark” personality characteristics (e.g., the “Dark Tetrad” of narcissism, Machiavellianism, psychopathy, and sadism), and more frequent use of alternative and fringe social media platforms. When examining a more comprehensive multivariate model, the variables that most strongly predict cryptocurrency ownership are being male, relying on alternative/fringe social media as one’s primary news source, argumentativeness, and an aversion to authoritarianism. These findings highlight numerous avenues for future research into the people who buy and trade cryptocurrencies and speak to broader global trends in anti-establishment attitudes and nonnormative behaviors.

## Introduction

Cryptocurrency (“crypto”) is an increasingly popular form of digital currency exchange and investing that is not produced, controlled, or supported by any central bank or government entity [1]. These currencies allow for both unregulated trading and anonymous transactions which has contributed, at least partially, to crypto markets that are both volatile and prone to fraud and failure [2, 3]. Given the volatility of crypto markets, potential for loss, and lack of regulatory oversight, many financial experts, as well as members of the public, view cryptocurrency as an unsound investment, with some even likening it to gambling or participation in a Ponzi scheme [4, 5]. Moreover, governments have expressed significant concern not only about cryptocurrency’s ability to bankrupt individuals and disrupt worldwide financial markets, but also its potential linkages to crime and terrorism [6].

While crypto ownership is not as prevalent as more established mainstream investments such as stocks, hundreds of millions of investors globally have entered this increasingly popular market [7, 8], spurring crypto assets to a collective value of more than three trillion USD in 2021 [9]. Despite the potential financial risks for individual investors, as well as recent large-scale collapses of cryptocurrency exchange companies and markets (e.g., FTX), many people continue to invest in and trade various cryptocurrencies, seeing them as an easy way to make quick money from investing [10].

Given the growing popularity of what many see as risky financial behavior [11], scholars have recently begun to probe the political, psychological, and social characteristics of cryptocurrency users to better understand the profile of people drawn to this type of investing. Some preliminary work suggests that individuals who have owned cryptocurrency differ from non-owners, in terms of both their political attitudes and identities and their psychological profiles [12, 13]. While the literature is continuing to grow, [14] it remains underdeveloped and is largely composed of studies that examine only a small number of factors in each given study and make use of using nonrepresentative samples. Given these limitations, and the potentially disruptive nature of crypto to both individuals and international financial markets, deeper, more exploratory and expansive investigations—including investigations of previous findings—are needed to provide crucial insight and offer a broader, more detailed view of people who invest in it. This need motivates our research question:

RQ: How are cryptocurrency buyers different from those who do not purchase cryptocurrency?

Here we provide results from a large national survey of 2,001 American adults, focusing on the associations between cryptocurrency ownership and a broad range of political, psychological, and social characteristics. We find that the variables most strongly correlated with cryptocurrency ownership are being male, having a victimhood mindset, and relying on alternative/fringe social media as one’s primary news source. These findings highlight numerous avenues for future research in this new and growing area of research and speak to broader global trends in anti-establishment attitudes and nonnormative behaviors.

## Hypotheses

With the advent of cryptocurrencies over the last decade, scholars have begun to shift more research attention to examining which attributes and behaviors are most strongly associated with buying and trading crypto [15, 16]. As cryptocurrencies are non-centralized and free from government intervention [17], it is perhaps unsurprising that people who invest or trade in cryptocurrencies prefer privacy in their financial transactions due to concerns over government monitoring [18]. Indeed, the motivation behind the development of cryptocurrency was rooted in a strong distrust for mainstream, traditional currencies and financial institutions [19]. A distrust of institutions and mainstream authorities is also strongly associated with characteristics such as conspiracy thinking, low levels of analytic and scientific reasoning, anti-science attitudes, the “need for chaos,” and nonmainstream political orientations [20–24], raising the question of whether these same attributes facilitate interest in cryptocurrency use [25]. Additionally, though cryptocurrency ownership has been linked to potentially harmful behavioural tendencies such problem gambling [26], its associations with other arguably negative proclivities, such as impulsiveness, paranoia, and various forms of aggression, are currently unknown.

Critically, existing research suggests that crypto investors display a heterogeneous collection of ideologies that defy easy categorization along a traditional, unidimensional left-right opinion space [13, 27]. For example, some scholars have noted streaks of libertarianism [28], populism [27], and anarchism [29] among investors. However, other studies have reported evidence of alt-right attitudes, far-right extremism, and white supremacist views [25, 30].

Taken together, this nascent profile of crypto investors is reflective of recent studies in which non-left-right political attitudes that express cynicism towards the establishment as a whole (e.g., conspiracy thinking, populist attitudes) are more predictive of certain nonnormative beliefs (e.g. voter fraud, QAnon) and behaviours (e.g., toxic online behaviours, engagement with extremist groups) than are traditional left-right attitudes and identities [20, 31–35]. We therefore suspect that cynical views towards the system as a whole will likely be related to the use of currencies that are outside the scope of the political system precisely because people holding those views will be more likely to engage in behaviours that are either outside of, or actively undermine, the established system [31, 35, 36]. This leads to our first four hypotheses:

H_1A_: Individuals who have purchased crypto currencies will display higher levels of conspiracism.
H_1B_: Individuals who have purchased crypto currencies will display lower levels of factors associated with analytic and scientific thinking.
H_2_: Individuals who have purchased crypto currencies will display a mixture of left- right political allegiances.
H_3_: Individuals who have purchased crypto currencies will display higher levels of populist sentiment.
H_4_: Individuals who have purchased crypto currencies will be more supportive of extremist groups.

Further, people who invest or trade in cryptocurrencies have been found to be more likely to share negative emotional states and nonnormative personality traits [37]. People who invest in cryptocurrencies report experiencing significantly higher levels of perceived anxiety, depression, impulsivity, loneliness, mood disorders, and stress compared to those who do not [38–40]. The extant literature also suggests that people who invest in cryptocurrencies display higher levels of nonnormative and “dark” personality traits [25].

H_5_: Individuals who have purchased crypto currencies will display higher levels of negative emotional affect and lower levels of positive emotional affect.
H_6_: Individuals who have purchased crypto currencies will display higher levels of nonnormative personality traits.

## Materials and method

Responses from 2,001 American adults (900 male, 1,101 female, *M*_age_ = 48.54, *SD*_age_ = 18.51, bachelor’s degree or higher = 43.58%) were collected from May 26 through June 30, 2022, by Qualtrics (qualtrics.com), who partnered with Cint and Dynata, to recruit a demographically representative sample (e.g., age, sex, education, income, race). All subject panels maintained by Cint and Dynata fully comply with European Society for Opinion and Marketing Research (ESOMAR) standards for protecting participant privacy and information security. Responses were excluded from any participants who failed six attention check items or who completed the survey in less than one-half of the estimated median completion time of 18.6 minutes (based on a “soft launch” pilot of n = 50). Respondents received incentives redeemable from the sample provider in exchange for their participation. Study approval was granted by the University of Miami Human Subject Research Office on May 13, 2022 (Protocol #20220472). Respondents read an informed consent letter and indicated their written agreement to participate by clicking the appropriate radio button displayed at the bottom of the screen (“Yes, I consent to participate” or “No, I do not consent to participate”). After providing consent, participants completed measures of the following variables:

### Dependent variable

Participants responded either “Yes” or “No” to the question: *“Do you currently*, *or have you ever*, *owned cryptocurrency*?*”*

### Independent variables

In addition to providing demographic information (age, sex, income, education level, religiosity), participants also responded to measures of various political (e.g., partisanship, ideology, non-left/right orientations), and psychological (e.g., personality traits, positive/negative affect, cognitive factors) characteristics as well as measures of respondents’ information environments and media consumption (e.g., watching local news, using social media, and the like), and their views towards science, experts, and authority. These items allow us to test our hypotheses and to explore a fuller profile of traits of crypto buyers. Full question wordings, rating scales, and descriptive statistics for all measures can be found in the S1 Appendix.

#### Conspiracism, thinking styles, and views towards science, experts, and authority

For factors related to thinking styles (such as analytic and scientific reasoning), we included measures of *patternicity* (3 items; α = .82) [41], *subjective numeracy* (3 items; α = .81) [42], *desire for simple solutions* (5 items; α = .79) [43], *intolerance of uncertainty* (5 items; α = .79) [44], and *conspiracy thinking* (5 items; α = .79) [35]. Respondents’ *scientific literacy* was measured by summing correct answers to six true/false general science questions (e.g., “Atoms are smaller than electrons”) [45]. We also assessed attitudes towards scientists and experts using two measures: (1) *anti-intellectualism* (7 items; α = .92) operationalized and measured as trust in experts (“Distrust a lot” to “Trust a lot”) across a broad range of STEM professions [46] and, (2) *confidence in the scientific community*, measured on a scale from “strongly disagree” to “strongly agree” using the single item, “I have confidence in the scientific community” [47].

Finally, to assess receptivity to epistemically suspect information, participants were presented with a list of 21 conspiracy theories and asked to indicate their endorsement of each by rating them from “strongly disagree” to “strongly agree.” Responses were re-coded such that “agree” and “strongly agree” represented *belief* in the conspiracy theory (coded as 1), while “don’t know,” “disagree” and “strongly disagree” reflected a *lack of belief* (0) in the conspiracy theory. A *total count of conspiracy theories believed* was calculated by summing the dichotomous (1 = belief; 0 = non-belief) responses to each conspiracy theory.

#### Political orientations and engagement

Participants rated themselves on measures of *partisanship* (Strong Democrat to Strong Republican) and *ideology* (very liberal to very conservative), *partisan intensity* and *ideological intensity* (i.e., the extremity of respondents’ ideology and partisanship without regard to left-right valence), *right-wing authoritarianism* (3 items; α = .68) [48], and *left-wing authoritarianism* (3 items; α = .90) [49]. We also included various measures of non-left/right political orientations such as *populism* (5 items; α = .79) [50], *national narcissism* (3 items; α = .80) [51], *gendered nationalism* (3 items; α = .86) [52, 53], *Christian nationalism* (4 items; α = .89) [54], *denialism* (4 items; α = .86) [55], and three single-item measures of trust: (1) *trust in government* (“The government can be trusted”), (2) *trust in police* (“The police can be trusted”), and (3) *trust in people* (“Most people are trustworthy”) [56].

We also gave respondents a standard battery to rate how qualified they feel to run for office, whether they think they might run for office one day and whether they believe someone like them can influence government (*political efficacy*) [57]. We also asked respondents a standard question about the extent to which they actively stay informed on politics and public affairs (*political interest*). Respondents also indicated the frequency with which they had engaged in specific political behaviors over the previous 12 months (a standard battery of behaviors including: contacted elected officials, volunteered during an election, and participated in political meetings, protests, civil disobedience, or acts of political violence) [57].

Finally, respondents used “feelings thermometers” to rate the strength of their attitudes toward several political figures and groups on a scale from 0 to 100, with scores below 50 reflecting negative feelings and scores above 50 reflecting positive feelings. The list of figures and groups includes several generally considered to be affiliated with the political left (Joe Biden, Bernie Sanders, Progressives, the Democrat Party), the political right (Donald Trump, the Republican Party), and those affiliated with more extreme political beliefs (Antifa, Vladimir Putin, QAnon, Proud Boys, White Nationalists).

#### Personality and motivational factors

To assess personality characteristics, respondents rated themselves on a broad battery of measures including *narcissism* (4 items; α = .89) [58], *Machiavellianism* (4 items; α = .88) [58], *psychopathy* (4 items; α = .87), *sadism* (4 items; α = .76) [59], *need for chaos* (6 items; α = .79) [20], *paranoia* (3 items; α = .91) [60], *schizotypy* (5 items; α = .87) [61], *dogmatism* (3 items; α = .69) [62], *argumentativeness* (3 items; α = .72) [35], *conflict escalation* in disagreements (6 items; α = .85) [63], *reactance* (4 items; α = .76) [64], *impulsiveness* (3 items; α = .74) [65], and *victimhood mindset* (4 items; α = .90). We also included two measures of goal orientation—*agentic goals* (5 items; α = .87) and *communal goals* (5 items; α = .87) [66]—and two measures of emotional experiences over the prior week, *positive affect* (10 items; α = .84) and *negative affect* (5 items; α = .88) [67].

#### Information environment

Respondents were presented with a list of 17 media sources (e.g., traditional news outlets, online news sites, social media sites) and rated how often they get “information about current events, public issues, or politics” from each [35]. The 17 media sources represented four distinct categories: (1) *legacy news media* (5 items; α = .79) includes traditional offline news sources such as network television news, cable news, local television, print newspapers, and radio; (2) *online mainstream news media* (3 items; α = .75) includes online news content (e.g., websites) from mainstream media sources such as mainstream newspaper websites, mainstream news magazines, and TV news websites; (3) *mainstream social media* (4 items; α = .80) includes YouTube, Facebook, Twitter, and Instagram; (4) *alternative social media* (5 items; α = .87) includes Reddit, Telegram, Truth Social, 8kun, and blogs.

### Method of analysis

All analyses were conducted using StataMP version 17.0 (stata.com).

## Results

### Prevalence of crypto ownership

**Fig 1:**
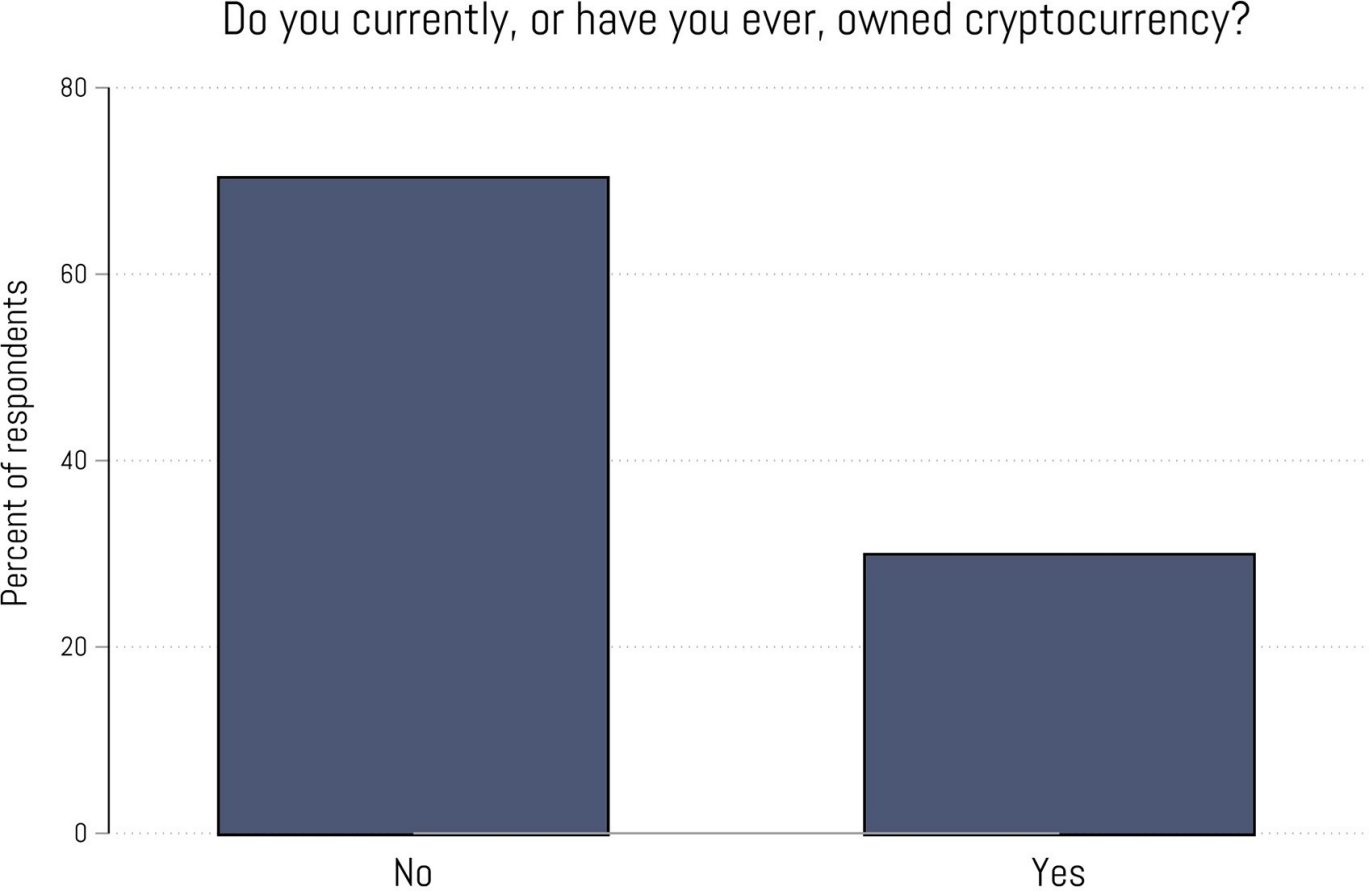
Distribution of responses (yes/no) to question asking if participants currently own or have owned cryptocurrency. Bars indicate the percentage of participants who selected each response. N = 2,001.

Fig 1 displays the percentage of participants who responded “Yes” and “No” to the question “Do you currently, or have you ever, owned cryptocurrency.” Just under 30% (29.79%; n = 596) answered “Yes” while 70.21% (n = 1405) answered “No.” Otherwise stated, these results suggest that nearly 77 million Americans have owned or currently own crypto. We now present a series of bivariate and multivariate relationships between crypto ownership and a wide range of respondent characteristics. Given the cross-sectional nature of our data, we make no claims regarding causation. Instead, our primary concern (at this early stage of the literature examining crypto ownership) is with elucidating a broad range of associations.

### Demographics

**Fig 2:**
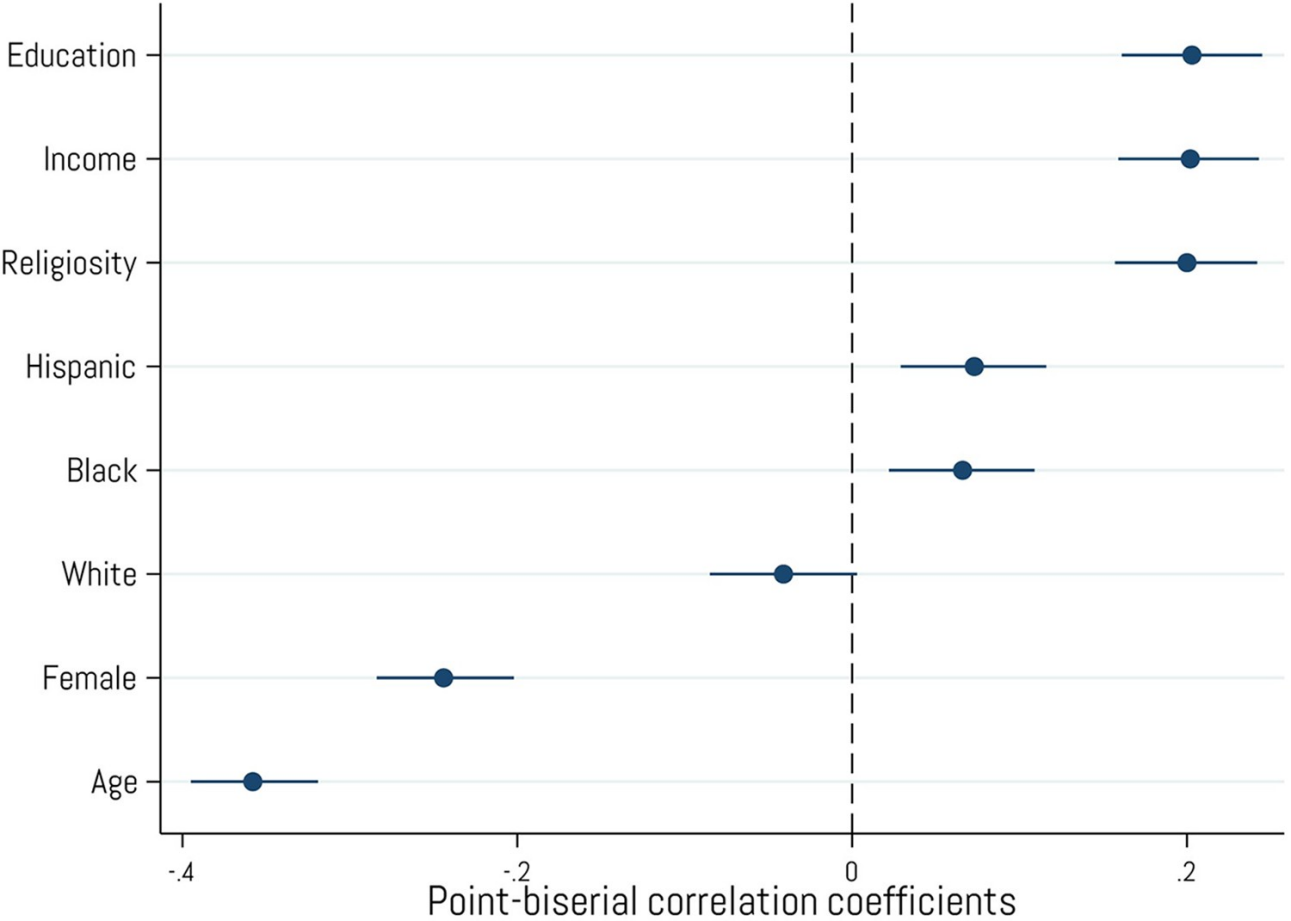
Associations with demographics variables. Point-biserial correlations between cryptocurrency ownership and various demographic categories. Error bars represent 95% confidence intervals. N = 2,001.

Respondents who own or have owned cryptocurrency were significantly younger (*M*_age_ = 38.38, *SD*_age_ = 12.94) than those who have not (*M*_age_ = 52.86, *SD*_age_ = 18.84), Welch’s *t*(1601.49) = 19.82, *p* < .001. As shown in Fig 2, cryptocurrency owners are largely male (*r* = -.24, *p* < .001), more educated (*r* = .20, *p* < .001), have a higher income (*r* = .20, *p* < .001), and are more religious (*r* = .20, *p* < .001) than those who have never owned cryptocurrency. Crypto ownership was also positively associated with respondents who identified as Black (*r* = .07, *p* < .001) and Hispanic (*r* = .07, *p* < .001), though the strength of those correlations was weak. Moreover, owning crypto was not significantly associated with respondents who identified as White (*r* = -.04, *p* = .065).

### Conspiracism, thinking styles and attitudes toward science

**Fig 3:**
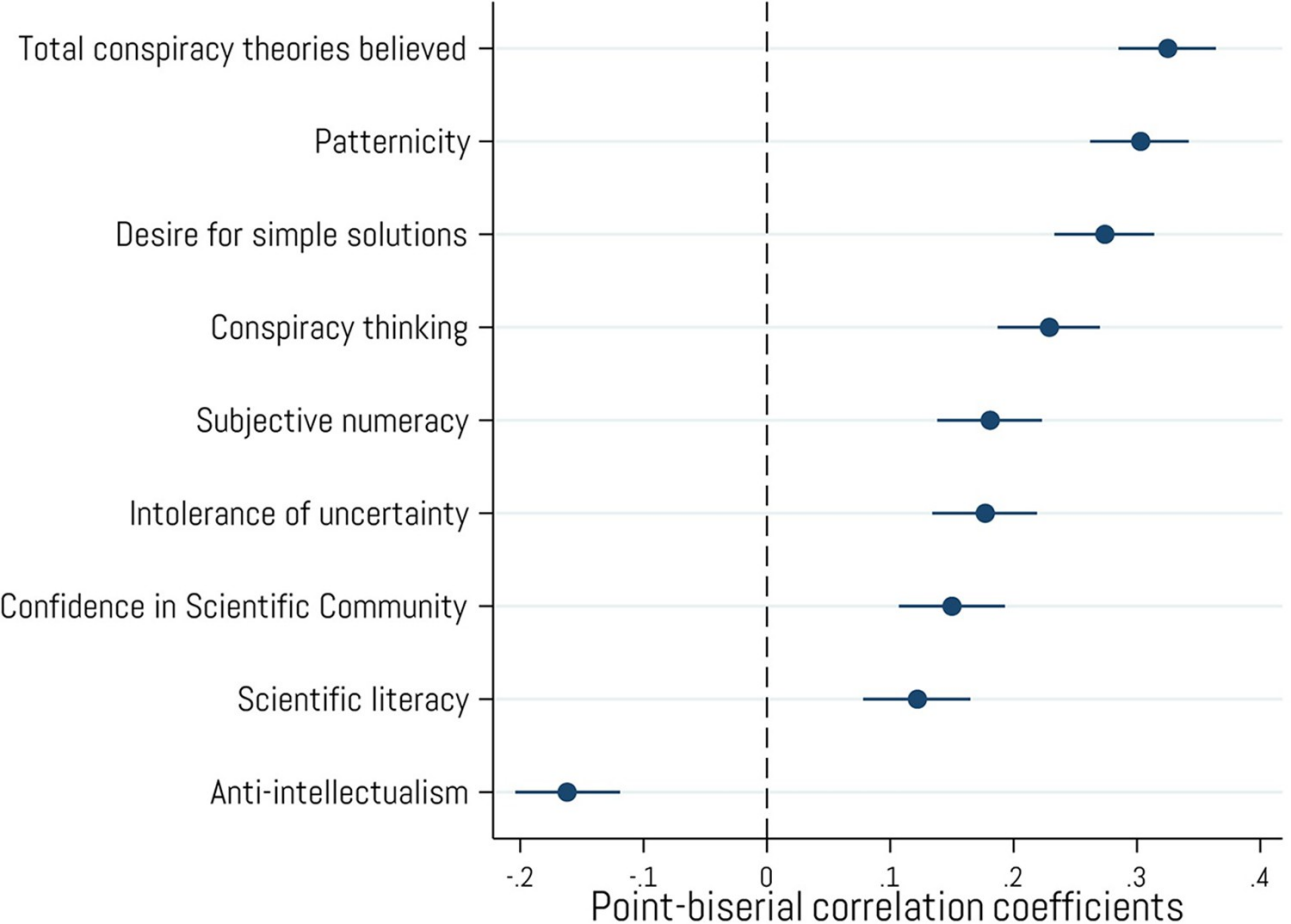
Associations with variables related to conspiracy thinking. Point-biserial correlations between cryptocurrency ownership and variables related to thinking styles, conspiracy beliefs, and attitudes toward science. Error bars represent 95% confidence intervals. N = 2,001.

Owning cryptocurrency is positively related to several variables that have been found to predict greater receptivity to epistemically weak claims and unwarranted beliefs (see Fig 3). For example, though crypto ownership is positively, though weakly, correlated with general scientific knowledge (*r* = .12, *p* < .001) and self-reported math ability (*r* = .18, *p* < .001), it is also more strongly associated with greater self-reported generalized pattern perception (*r* = .30, *p* < .001), higher levels of a conspiratorial thinking style (*r* = .23, *p* < .001), and belief in a greater number of conspiracy theories (*r* = .33, *p* < .001). Such findings may seem, at first glance, to be at odds with our other correlational results showing that crypto ownership is weakly associated with overall confidence in the scientific community (*r* = .12, *p* < .001) and less distrust of experts (*anti-intellectualism*, *r* = -.16, *p* < .001). However, the fact that cryptocurrency owners are more conspiracy-minded while also being somewhat more trustful and confident in scientific experts may be explained by their increased intolerance of uncertainty (*r* = .18, *p* < .001) and a greater desire for problems to have simple, easy solutions (*r* = .27, *p* < .001). Our findings therefore support H_1_, that individuals who have purchased crypto currencies will display higher levels of factors commonly associated with conspiracism.

### Left/right political orientations

**Fig 4:**
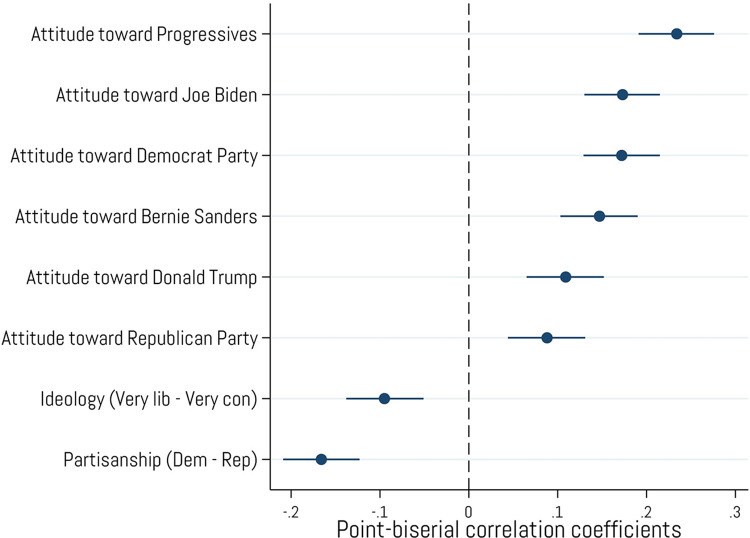
Associations with left/right political orientations and attitudes. Point-biserial correlations between cryptocurrency ownership and ratings of traditional left/right political orientations and “feeling thermometer (0–100) ratings of various political figures and groups. Error bars represent 95% confidence intervals. N = 2,001.

When examining classic left/right political orientations and attitudes (Fig 4), we found that crypto owners tend to be marginally more liberal in their political views (*r* = -.10, *p* < .001) and somewhat more likely to identify as Democrats (*r* = -.17, *p* < .001). We also found positive correlations between crypto ownership and feeling thermometer ratings of various well-known political figures and groups, such as Progressives (*r* = .23, *p* < .001), Joe Biden (*r* = .17, *p* < .001), the Democratic Party (*r* = .17, *p* < .001), and Bernie Sanders (*r* = .15, *p* < .001). Ownership was also positively, though weakly, related to ratings of Donald Trump (*r* = .11, *p* < .001) and the Republican Party (*r* = .09, *p* < .001). We note that some of the correlations reported here are statistically significant, but small in magnitude [68] and may simply reflect natural variation in our sample. We therefore urge caution when interpreting these results with regard to whether crypto ownership is meaningfully associated with traditional left/right political views. Regardless, and taken as a whole, these inconsistent findings suggest that crypto purchasers display a mixture of left- right political allegiances, providing support for H_3_.

### Non-left/right political orientations

**Fig 5:**
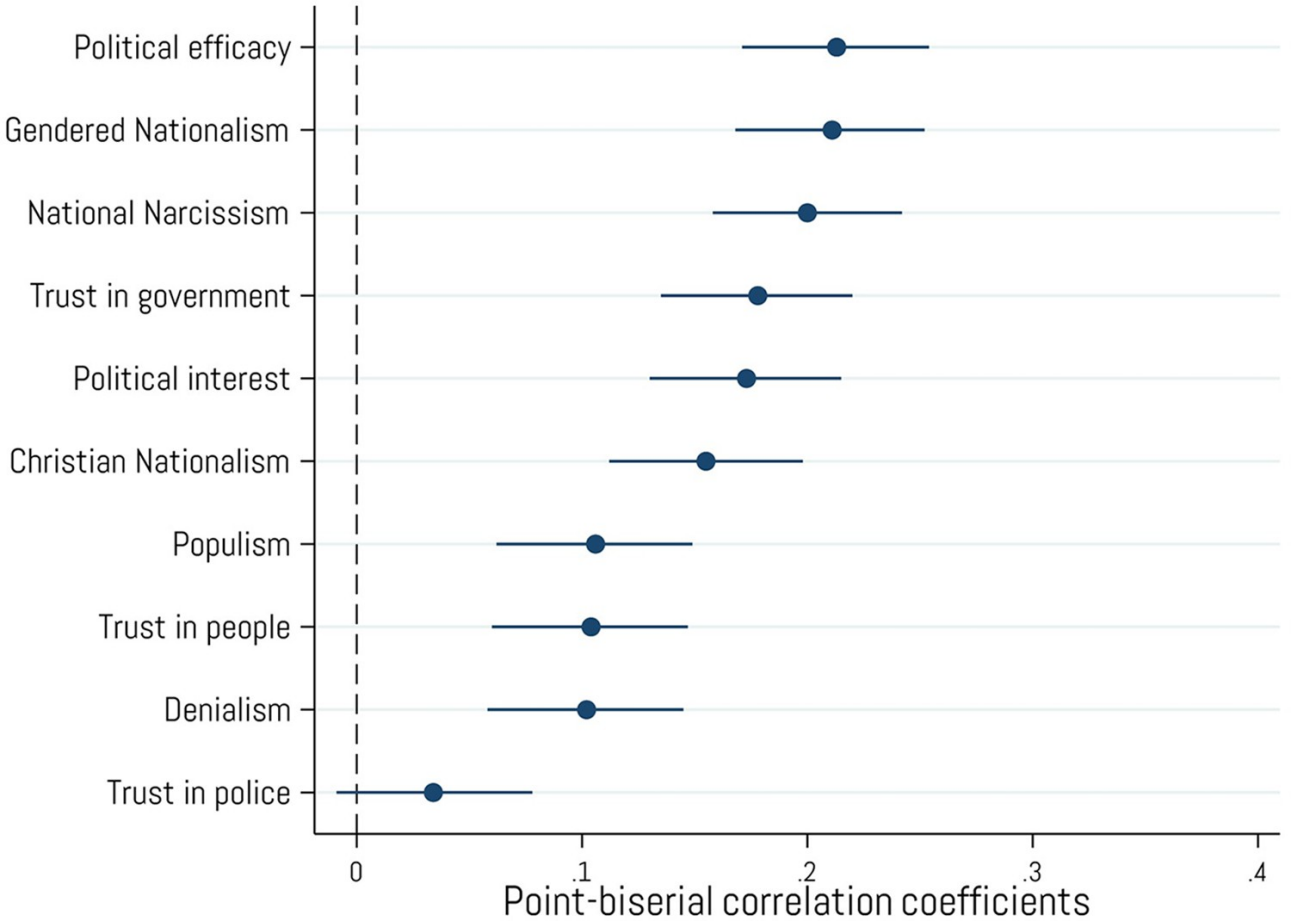
Associations with non-left/right political orientations and attitudes. Point-biserial correlations between cryptocurrency ownership and ratings of non-left/right political orientations and “feeling thermometer (0–100) ratings of various political figures and groups. Error bars represent 95% confidence intervals. N = 2,001.

Owning cryptocurrency is positively associated with various political orientations that fall outside traditional left/right categorizations (see Fig 5). For instance, crypto ownership is positively correlated with beliefs in American exceptionalism (*national narcissism*; *r* = .20, *p* < .001), beliefs that the U.S. should be more masculine (*gendered nationalism*; *r* = .21, *p* < .001), and beliefs that the U.S. should be governed by Christian values (*Christian nationalism*; *r* = .16, *p* < .001). Additionally, crypto ownership is positively associated with *trust in the government* (*r* = .18, *p* < .001), staying informed on politics and current events (*political interest*; *r* = .17, *p* < .001), and the belief that one can influence political processes at an individual level (*political efficacy*; *r* = .21, *p* < .001). Finally, crypto ownership is not strongly related to *populism*, *trust in people*, or *denialism* (all *r* ≤ .11, all *p* < .001) and is unrelated to *trust in the police*. Our finding regarding populism supports H_3_ in that individuals who have purchased cryptocurrencies will display higher levels of populist sentiment. More generally, individuals who have purchased cryptocurrencies display stronger non-left-right political attitudes.

### Left/right political extremity

**Fig 6:**
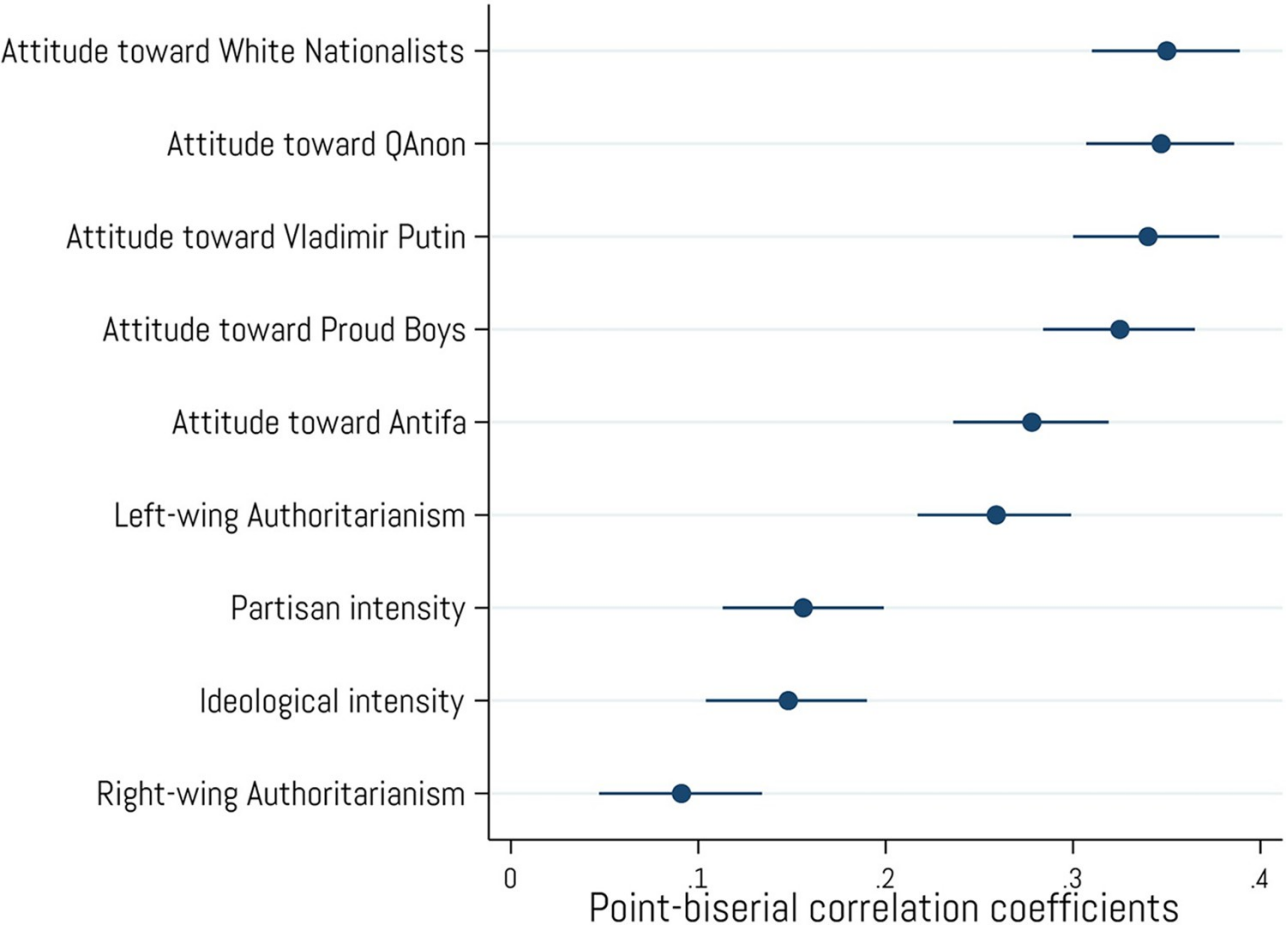
Associations with left/right political orientations and attitudes. Point-biserial correlations between cryptocurrency ownership and ratings of traditional left/right political orientations and “feeling thermometer (0–100) ratings of various political figures and groups. Error bars represent 95% confidence intervals. N = 2,001.

We next examined whether crypto ownership is associated with more extreme political attitudes (Fig 6), finding positive correlations with feeling thermometer ratings of White Nationalists (*r* = .35, *p* < .001), QAnon (*r* = .35, *p* < .001), Vladimir Putin (*r* = .34, *p* < .001), and Proud Boys (*r* = .33, *p* < .001). Crypto ownership was also positively associated with ratings of Antifa (*r* = .28, *p* < .001) and left-wing authoritarian beliefs (*r* = .26, *p* < .001), but was only weakly associated with right-wing authoritarianism (*r* = .06, *p* < .001). Finally, crypto ownership shared small, but significant, associations with the intensity/extremity of respondents’ partisan (*r* = .16, *p* < .001) and ideological leanings (*r* = .15, *p* < .001). These results support H_4_, as individuals who have purchased cryptocurrencies are more supportive of extremist groups and are somewhat more likely to identify on the extremities of partisanship and ideology.

### Interpersonal and political behaviors

**Fig 7:**
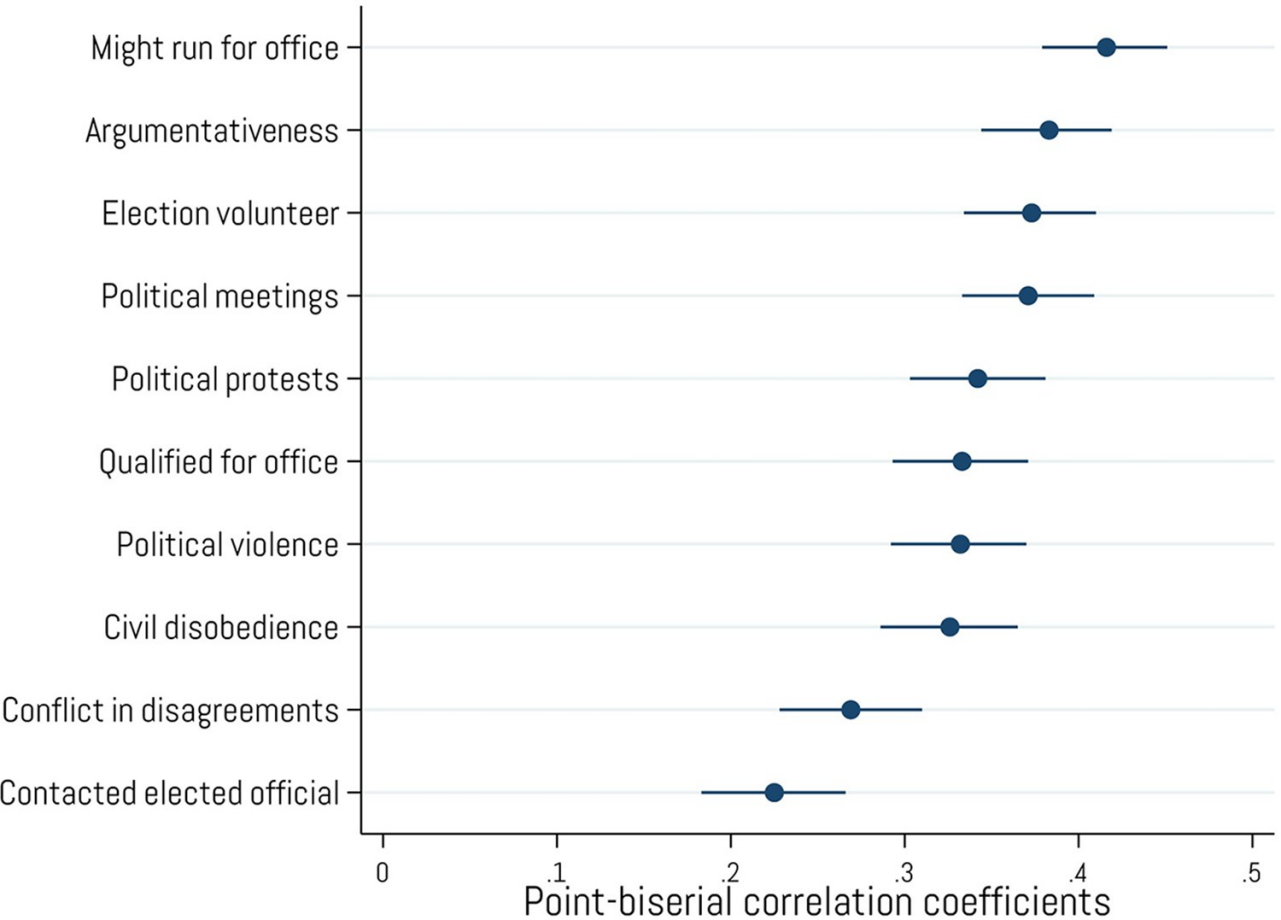
Associations with interpersonal and political behaviors. Point-biserial correlations between cryptocurrency ownership and various interpersonal and political behaviors. Error bars represent 95% confidence intervals. N = 2,001.

We also examined the extent to which crypto owners participate in both normative and nonnormative political activities and how they interact with others (see Fig 7). For instance, crypto ownership is positively related to the belief that one is qualified to hold public political office (*r* = .33, *p* < .001) and to the desire to one day run for office (*r* = .42, *p* < .001). Crypto ownership is also positively related to various forms of normative civic engagement such as volunteering during an election (*r* = .37, *p* < .001), attending political meetings (*r* = .37, *p* < .001) and protests (*r* = .34, *p* < .001), engaging in civil disobedience (*r* = .33, *p* < .001), and contacting elected officials (*r* = .23, *p* < .001). However, crypto ownership is also positively related to more problematic forms of interpersonal and political activity such as being argumentative (*r* = .38, *p* < .001), escalating conflict when disagreeing with others (*r* = .27, *p* < .001), and taking part in political violence (*r* = .33, *p* < .001).

### Personality, emotional, and motivational attributes

Crypto ownership is positively associated with several so-called “dark” personality characteristics including narcissism (*r* = .38, *p* < .001), Machiavellianism (*r* = .29, *p* < .001), psychopathy (*r* = .27, *p* < .001), and sadism (*r* = .27, *p* < .001). Relatedly, crypto owners also tend to score higher on measures of need for chaos (*r* = .35, *p* < .001), paranoia (*r* = .35, *p* < .001), schizotypal attributes (*r* = .35, *p* < .001), dogmatism (*r* = .23, *p* < .001), victimhood mentality (*r* = .18, *p* < .001), and psychological reactance (*r* = .11, *p* < .001). Crypto owners also indicated that agentic goals related to success, status, and achievement (*r* = .31, *p* < .001) are more important to them than communal goals which involve helping others (*r* = .08, *p* < .001).

**Fig 8:**
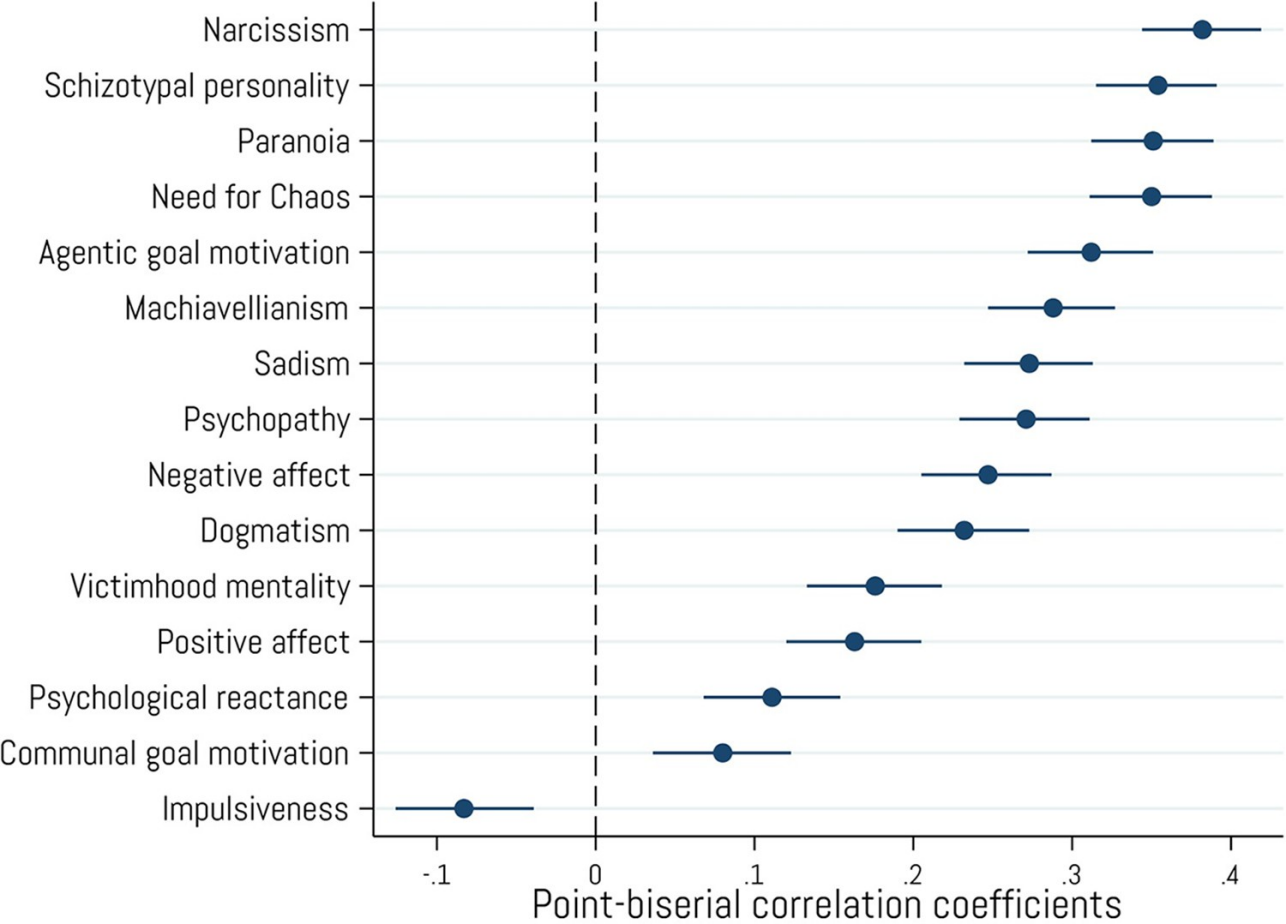
Associations with personality, emotional, and motivational attributes. Point-biserial correlations between cryptocurrency ownership and personality, emotional, and motivational variables. Error bars represent 95% confidence intervals. N = 2,001.

Finally, crypto ownership is more strongly related to experiencing negative emotions (*r* = .25, *p* < .001) than positive (*r* = .16, *p* < .001). See Fig 8 for a full list of correlations with these variables. These findings first show partial support for H_5_, whereby crypto owners will display higher levels of negative emotional affect; however, these same individuals also show higher levels of positive emotional affect. These results also show support for H_6_, specifically that purchasers of crypto currencies display higher levels of nonnormative personality traits, including dark tetrad traits.

### Media use

**Fig 9:**
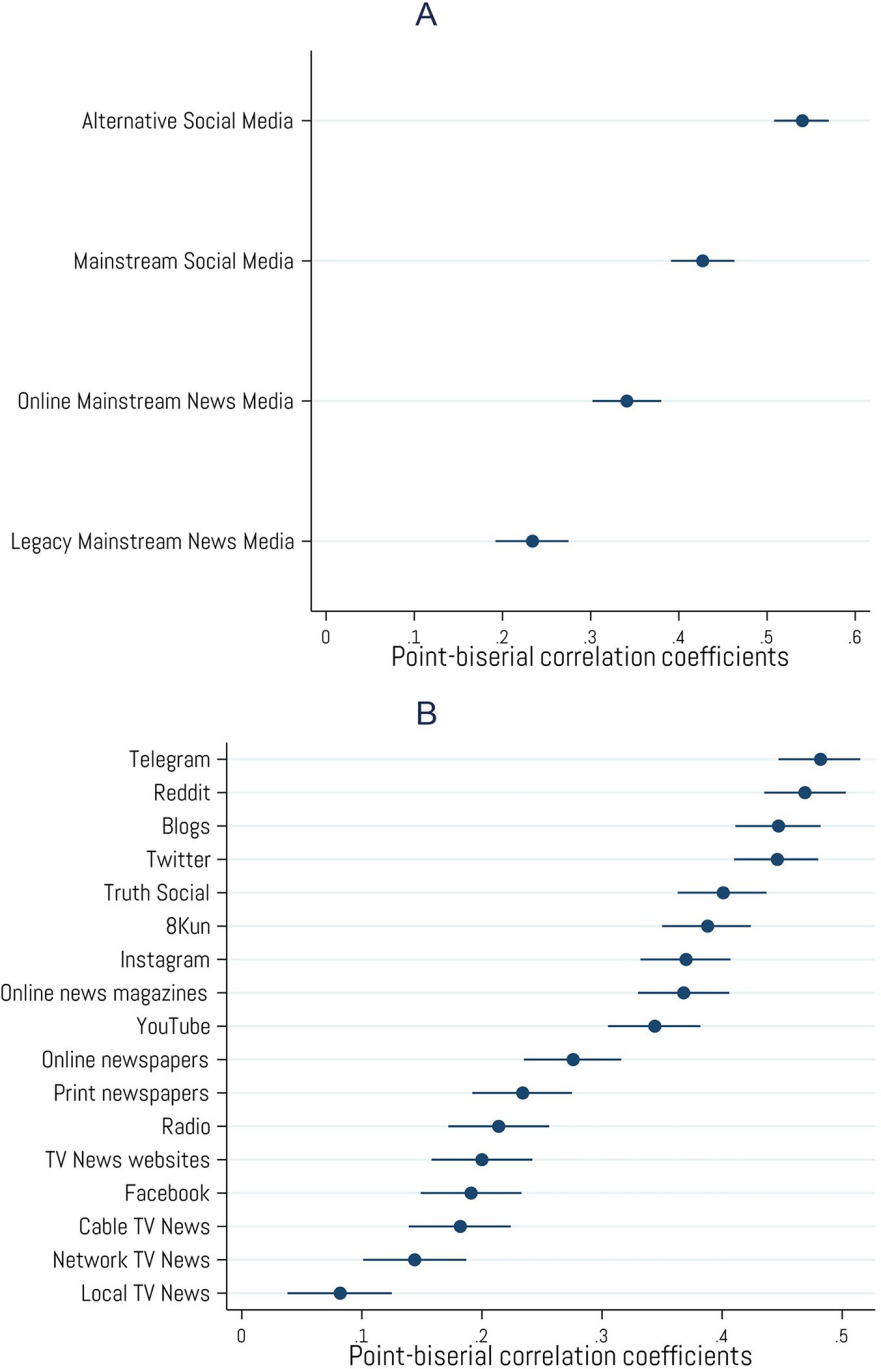
Associations with information environment. Point-biserial correlations between cryptocurrency ownership and various online and offline group (A) and individual (B) sources of news information. Error bars represent 95% confidence intervals. N = 2,001.

Cryptocurrency ownership is strongly associated with getting news about current events, public issues, and politics from *alternative social media sources* (*r* = .54, *p* < .001) such as Telegram (*r* = .48, *p* < .001), Reddit (*r* = .47, *p* < .001), blogs (*r* = .45, *p* < .001), Truth Social (*r* = .40, *p* < .001), and 8Kun (*r* = .39, *p* < .001). Crypto ownership is also moderately correlated with getting news from more *mainstream social media sources* (*r* = .43, *p* < .001) such as Twitter (*r* = .45, *p* < .001) and Instagram (*r* = .37, *p* < .001), YouTube (*r* = .34, *p* < .001) and, to a lesser extent, Facebook (*r* = .19, *p* < .001). Crypto ownership was less strongly associated with getting one’s news from more traditional *legacy news media sources* whether they be online (*r* = .34, *p* < .001) or offline (*r* = .23, *p* < .001). A full list of associations with media sources can be found in Fig 9.

### Multivariate analysis of cryptocurrency ownership

**Fig 10:**
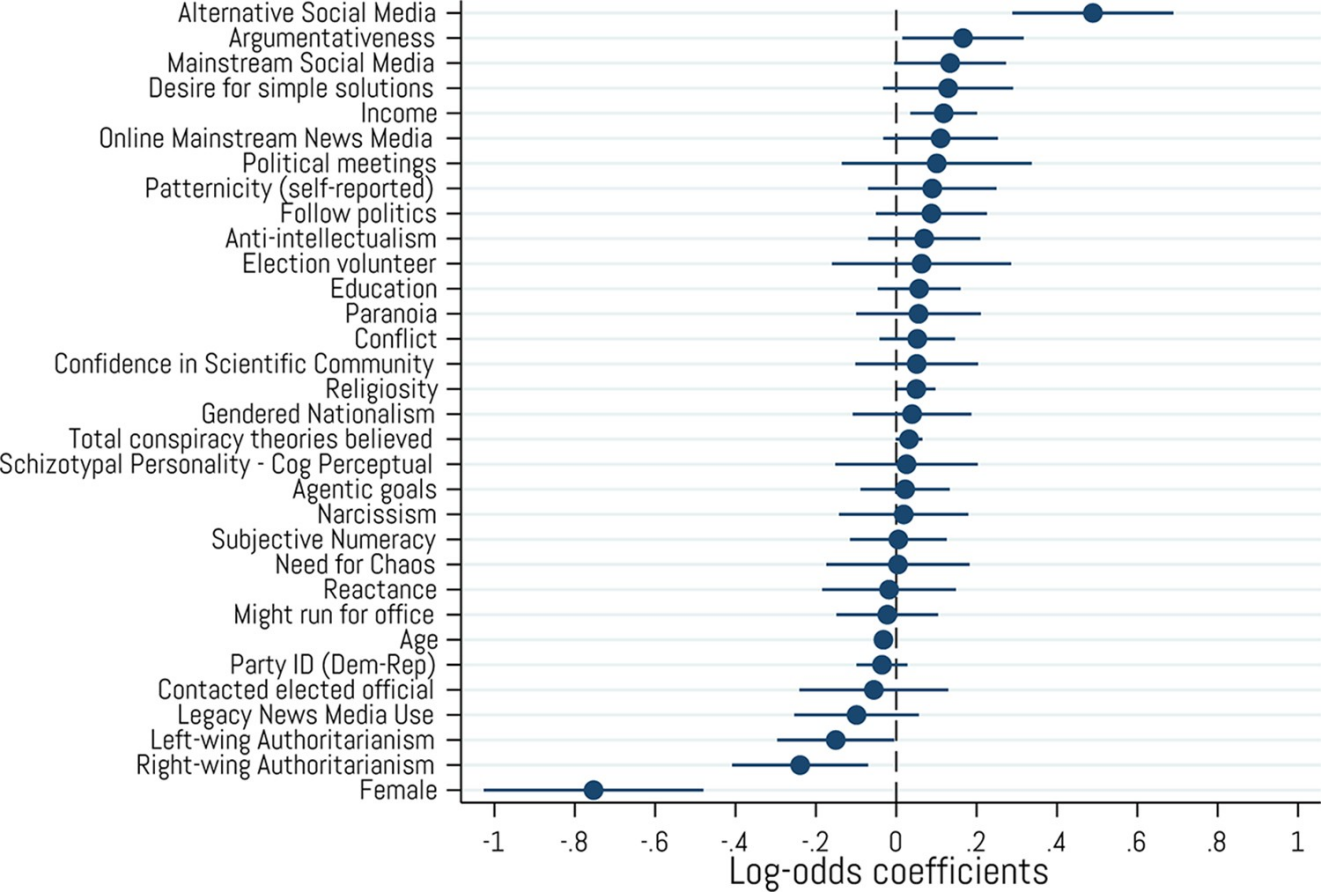
Logistic regression predicting cryptocurrency ownership from demographics, thinking styles and intellectual attitudes, personality characteristics, and media environment. Coefficients are log odds. Error bars represent 95% confidence intervals. N = 2,001.

Based on the results reported above, we conducted a final exploratory investigation by constructing a comprehensive multivariate logistic regression model to predict cryptocurrency ownership (Fig 10). We first conducted three logistic regression analyses predicting cryptocurrency ownership from each group of variables reported earlier (i.e., conspiracism and thinking styles, political alignment and behaviors, and political and motivational factors). To avoid redundancy and construct overlap in the regression model for political variables, we excluded all “feeling thermometer” variables that measured attitudes toward partisan/ideological elites and political groups. From these results (see S1 Appendix), we selected our predictors for the final exploratory investigation from the significantly correlated demographic categories (gender, age, education, income, and religiosity), as well as all significant predictors from the preliminary regression analysis of each variable group (i.e., conspiracism and thinking styles, political alignment, personality/motivational characteristics, and media use). The results show that cryptocurrency ownership is most strongly predicted by being male or male-identifying (*ˆβ* = -.75, *p* < .001) and relying on alternative or fringe online social media sources for one’s news information about current events, public issues, and politics (*ˆβ* = .49, *p* < .001). Crypto ownership was also negatively predicted by both types of authoritarianism (right-wing: (*ˆβ* = -.24, *p* = .006; left-wing: (*ˆβ* = -.15, *p* = .043) and significantly, but less strongly, predicted by argumentativeness (i.e., one’s enjoyment of and willingness to argue with others over a range of topics) (*ˆβ* = .17, *p* = .031), having higher income (*ˆβ* = .12, *p* = .005), and religiosity (*ˆβ* = .05, *p* = .040). Finally, frequency of using mainstream social media (*ˆβ* = .13, *p* = .060) and the total number of conspiracy theories a person believes (*ˆβ* = .03, *p* = .063) both marginally predicted cryptocurrency ownership, though this latter association was markedly weak. No other covariates included in the model significantly predicted cryptocurrency ownership.

**Fig 11:**
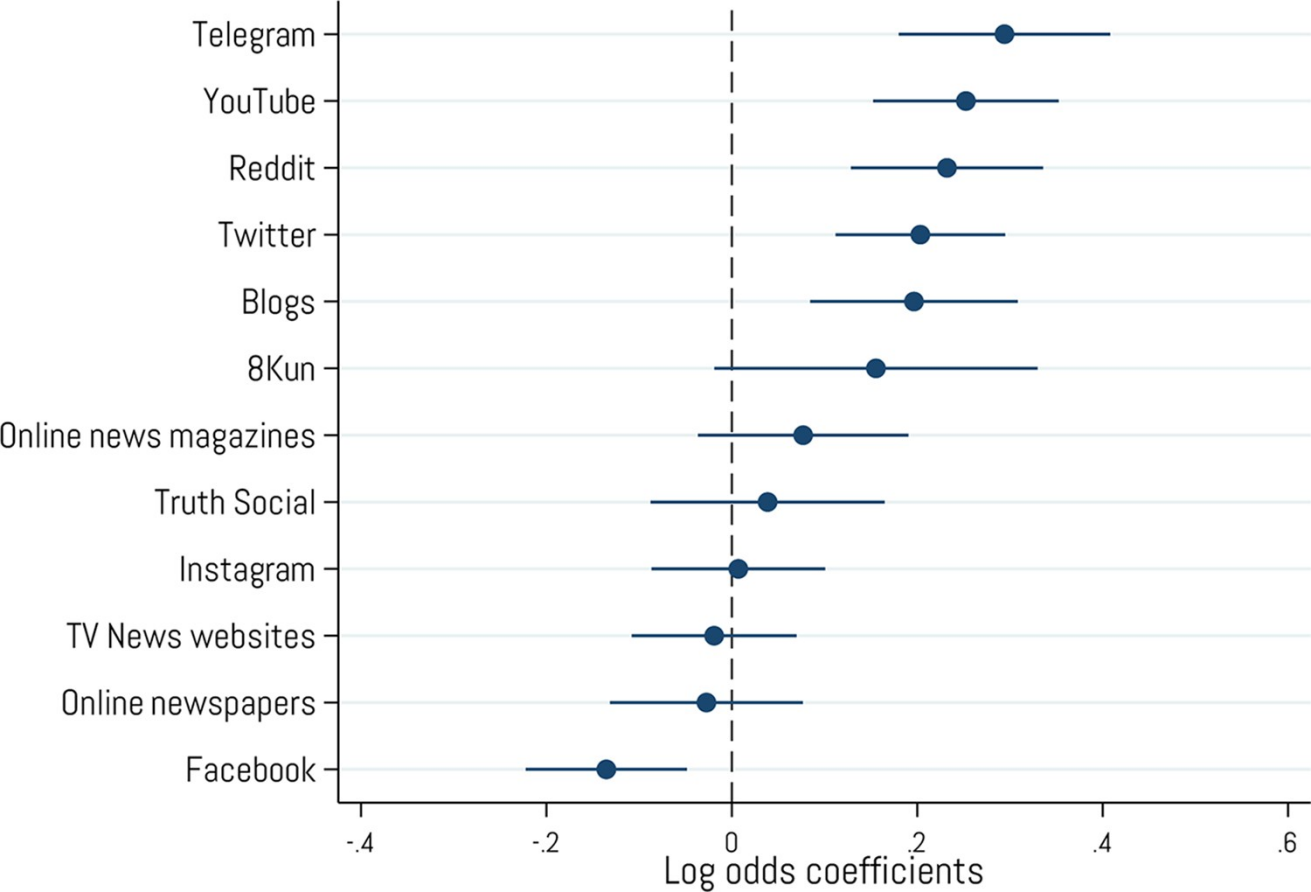
Logistic regression predicting cryptocurrency ownership from individual online media sources. Coefficients are log odds. Error bars represent 95% confidence intervals. N = 2,001.

Given that reliance on alternative or fringe social media sources for news information was the strongest positive predictor of cryptocurrency ownership in our multivariate analysis, we were also curious which specific outlets or media types from this category showed the largest association with crypto ownership. Therefore, as a final exploratory investigation, we constructed a multivariate logistic regression model predicting crypto ownership from individual online media sources (Fig 11). The results show that the strongest predictors among online media sources were Telegram, YouTube, Reddit, Twitter, and blogs. Interestingly, the only online media news source that negatively predicted crypto ownership was Facebook.

## Conclusion

A product of the internet age, crypto allows people to buy, sell, invest, and trade without the help of government currencies or oversight. At the same time, cryptocurrency markets have been volatile and prone to various forms of fraud. Recent events, as well as its unregulated nature, suggest that the freedom and privacy afforded by crypto currencies come with costs and risks [69]. Indeed, these issues may be key reasons why 75% of U.S. citizens currently view cryptocurrencies as financially unsafe and unreliable [11]. Crucially, the results presented here suggest that cryptocurrency ownership is associated with several nonnormative and arguably maladaptive characteristics.

Specifically, at the bivariate level we found that individuals who had purchased crypto were, on average, more likely to believe conspiracy theories, support extremist groups, and share populist sentiments. Further, crypto owners in our data set were more likely to display a range of left-right political identities that defy easy categorization. In addition, crypto purchasers were likely to have elevated levels of both positive and negative affect, as well as nonnormative personality traits (e.g., dark tetrad traits). Finally, crypto purchasers were likely more likely to use fringe sources of information as opposed to more mainstream outlets of news. When considering all relevant variables representing demographics, thinking styles and intellectual attitudes, personality characteristics, and media environments, a profile emerged reflecting cryptocurrency owners as more likely to be male or male-identifying, have somewhat higher income, who feel victimized by a life they perceive as having been unfair, and tend to get their news information about politics, public issues, and current events from social media sources such as Telegram, YouTube, Reddit, Twitter posts, and blogs.

By using a large national sample to compare crypto ownership to a wide range of individual level political, psychological, and social characteristics, we have not only provided much needed information about crypto purchasers but have illuminated numerous avenues for future research. For example, while the observational data presented here expand our understanding of the characteristics of cryptocurrency owners, future studies would benefit from an experimental approach to assess whether the correlations identified here are casual. More specifically, given the strong relationship between media use and cryptocurrency ownership we have identified, do different modalities (e.g., online versus traditional), rhetorical forms (e.g., framed as a more private and decentralized form of investment versus as a chance for quick and easy profit), and sources (e.g., celebrity and other forms of endorsement) of communication influence the desire to buy cryptocurrency? If they are indeed causal in nature, future studies should also examine whether these types of experimental effects vary across individuals (e.g., are people with stronger non-normative personality traits more persuaded by the privacy and decentralization of cryptocurrency, or the prospect of quick and easy profit?).

Additionally, future studies could expand on these results by examining the public’s attitudes on this new form of currency more broadly. More specifically, here we have used a simple yes/no indicator of cryptocurrency ownership; future studies could make use of a more well-developed multi-item scale assessing attitudes on cryptocurrency ownership in addition to past, current, and future intent to own. Likewise, future studies could delineate between different types of cryptocurrency ownership (e.g., are owners of the more mainstream Bitcoin different from owners of nascent cryptocurrencies?). Further, researchers might concentrate on the factors that make cryptocurrencies attractive to potential investors: is it their distrust of particular financial institutions or perhaps a desire to realize quick financial gains, for example?

Finally, our results here suggest that crypto purchasers, on average, share an eclectic mix of political attitudes, identities, and predispositions. This finding contrasts with past claims that crypto currencies are some sort of financial shibboleth of the “far-right” [70]. Future work would benefit from more deeply investigating possible connections between the motivations underlying cryptocurrency ownership (e.g., privacy concerns, avoiding government oversight, skirting inflationary government currencies) and specific partisan worldviews and ideologies. As governments seek to more tightly regulate, or in some cases, emulate, cryptocurrencies, understanding the attraction of such currencies will be necessary. More work in this area is necessary and our results here should not be taken as a final word. Studies employing time-series data and experiments could improve on our correlative findings, and future work should examine how the base of crypto owners change over time as the currency becomes more (or less) accepted in society.

Taken together, the results presented here and the future research avenues they might speak to broader global trends in distrust, populism, and cynicism of established institutions [71–75], as well as their nonnormative behavioural and society-wide consequences [76, 77]. We are in era where established authorities are under attack, be they political institutions or financial markets [31, 35, 36, 77–79]. Both can lead to negative externalities (i.e., economic instability) [78, 80, 81]. As such, understanding their causes is of vital importance.

## Supporting information

S1 Appendix Supporting information. This is the file for the supporting information.
(DOCX)
