# Supplementary material for: The political, psychological, and social correlates of cryptocurrency ownership
Source: PLoS One. 2024 Jul 3;19(7):e0305178. doi: 10.1371/journal.pone.0305178 (PMC11221751; doi:10.1371/journal.pone.0305178)
Supplement: S1 Appendix — This is the file for the supporting information. (DOCX) [file pone.0305178.s001.docx]

## Appendix: Correlates of cryptocurrency investors

| **Table of Contents** | |
| --- | --- |
| Section | Page |
| A1. Demographic Items and Sample Composition | 2 |
| A2. Descriptive Statistics and Item Wordings for all variables | 4 |
| A3. Finding #2: Correlations with demographic variables | 14 |
| A4. Finding #3: Thinking styles and attitudes toward science | 14 |
| A5. Finding #4: Left-right political orientations | 15 |
| A6. Finding #5: Non-Left/Right Political Orientations | 15 |
| A7. Finding #6: Political extremity | 16 |
| A8. Finding #7: Interpersonal and political behaviors | 16 |
| A9. Finding #8: Personality, emotional, and motivational characteristics | 17 |
| A10. Findings #9a and #9b: Media Use by General Type | 18-19 |
| A11. Logistic regression predicting cryptocurrency ownership | 20 |
| A12. Logistic regressions by variable group | 21 |
| A13. Cryptocurrency ownership and conspiracy theory belief | 23 |
| A14. Appendix References | 24 |

**A1. Demographic Items and Sample Composition**

*Sex*

I am:

- Male
- Female

*Race*

I am: (please check all that apply)

- White
- Black or African-American
- Asian-American or Pacific Islander
- Native American or American Indian
- Other

*Income*

What is your annual household income?

- $24,999 or less
- $25,000 to $49,999
- $50,000 to $74,999
- $75,000 to $99,999
- $100,000 to $149,999
- $150,000 to $199,999
- $200,000 or more

*Education*

What is the highest level of education you have completed?

- Less than high school
- High school graduate or GED
- Some college, but no degree (yet)
- 2-year college degree
- 4-year college degree
- Post-graduate degree (MA, MBA, MD, JD, PhD, etc.)

*Age (Calculated from Birth Year)*

What year were you born?

| **Table A1. Sample Demographics** | | |
| --- | --- | --- |
|  | *Survey sample* | *U.S. Census*  *(age 18+)* |
| Sex^1^ |  |  |
| male | 45.0% | 49.1 |
| female | 55.0 | 50.9 |
| Education^2^ |  |  |
| some high school or less | 2.9 | 10.0 |
| high school graduate/GED | 24.3 | 28.0 |
| some college | 18.4 | 17.0 |
| 2-year degree | 10.8 | 10.0 |
| college graduate | 25.8 | 22.0 |
| Graduate Degree | 17.7 | 13.0 |
| Household Income^3^ |  |  |
| $24,999 or less | 18.8 | 18.1 |
| $25,000 to $49,999 | 20.4 | 19.7 |
| $50,000 to $74,999 | 16.6 | 16.5 |
| $75,000 to $99,999 | 12.3 | 12.2 |
| $100,000 to $149,999 | 14.4 | 15.3 |
| $150,000 to $199,999 | 7.7 | 8.0 |
| $200,000 or more | 9.9 | 10.3 |
| Race^1^ |  |  |
| White, non-Hispanic | 67.7 | 62.6 |
| Black, non-Hispanic | 13.5 | 12.2 |
| Asian, non-Hispanic | 2.7 | 2.8 |
| Native American, non-Hispanic | 1.9 | 0.7 |
| Hispanic or Latino origin | 16.2 | 16.6 |
| Age^1^ |  |  |
| 18 to 24 years | 7.9 | 11.7 |
| 25 to 44 years | 42.6 | 34.4 |
| 45 to 64 years | 19.4 | 32.8 |
| 65 years and over | 30.2 | 21.1 |
| U.S. Census Sources:  ^1^https://www.census.gov/data/tables/time-series/demo/popest/2020s-national-detail.html  ^2^https://www.census.gov/data/tables/2021/demo/educational-attainment/cps-detailed-tables.html  ^3^https://www.census.gov/data/tables/time-series/demo/income-poverty/cps-hinc/hinc-01.html | | |

**A2. Descriptive Statistics and Item Wordings for all variables**

| **Table A2. Descriptive Statistics** | | | | | |
| --- | --- | --- | --- | --- | --- |
|  | **N** | **Mean** | **Std. Dev.** | **Min** | **Max** |
| Own/owned cryptocurrency | 2001 | 0.30 | 0.46 | 0 | 1 |
| Gender (Female) | 2001 | 0.55 | 0.50 | 0 | 1 |
| Race (White) | 2001 | 0.78 | 0.41 | 0 | 1 |
| Income | 2001 | 3.46 | 1.93 | 1 | 7 |
| Education | 2001 | 3.86 | 1.54 | 1 | 6 |
| Age | 2001 | 48.54 | 18.52 | 19 | 95 |
| Religiosity | 2000 | 0.54 | 0.32 | 0 | 1 |
| Party ID (Dem-Rep) | 2001 | 3.49 | 2.24 | 1 | 7 |
| Ideology (Lib-Con) | 2000 | 3.88 | 1.84 | 1 | 7 |
| Partisan intensity | 2001 | 3.00 | 1.12 | 1 | 4 |
| Ideological intensity | 2000 | 2.39 | 1.22 | 1 | 4 |
| Right-Wing Authoritarianism | 2000 | 3.16 | 0.87 | 1 | 5 |
| Left-Wing Authoritarianism | 2001 | 2.76 | 0.93 | 1 | 5 |
| Political interest (follows politics) | 2000 | 3.66 | 1.12 | 1 | 5 |
| Political efficacy |  |  |  |  |  |
| Trust in government | 1999 | 2.84 | 1.20 | 1 | 5 |
| Trust in police | 2000 | 3.35 | 1.18 | 1 | 5 |
| Trust in “most people” | 2001 | 3.22 | 1.15 | 1 | 5 |
| National narcissism | 2001 | 3.06 | 1.02 | 1 | 5 |
| Gendered nationalism | 1999 | 2.97 | 1.16 | 1 | 5 |
| Christian Nationalism | 2001 | 2.99 | 1.01 | 1 | 5 |
| Populism (anti-establishment) | 2000 | 3.64 | 0.79 | 1 | 5 |
| Denialism | 2001 | 3.33 | 0.88 | 1 | 5 |
| Argumentativeness | 1999 | 2.86 | 1.06 | 1 | 5 |
| Conflict | 1993 | 0.81 | 1.46 | 0 | 6 |
| Need for Chaos | 2000 | 2.11 | 1.06 | 1 | 5 |
| Narcissism | 1999 | 2.56 | 1.08 | 1 | 5 |
| Machiavellianism | 2001 | 2.26 | 1.06 | 1 | 5 |
| Psychopathy | 2000 | 2.27 | 1.00 | 1 | 5 |
| Sadism | 2000 | 2.67 | 0.98 | 1 | 5 |
| Paranoia | 2000 | 2.06 | 1.13 | 1 | 5 |
| Schizotypy | 1999 | 2.72 | 1.07 | 1 | 5 |
| Psychological reactance | 2001 | 3.48 | 0.83 | 1 | 5 |
| Dogmatism | 2000 | 3.14 | 0.90 | 1 | 5 |
| Victimhood | 2000 | 2.78 | 1.07 | 1 | 5 |
| Impulsiveness | 2001 | 2.05 | 0.69 | 1 | 5 |
| Patternicity | 1999 | 3.20 | 0.99 | 1 | 5 |
| Uncertainty intolerance | 2001 | 3.13 | 1.98 | 1 | 5 |
| Subjective numeracy | 2001 | 4.29 | 1.30 | 1 | 6 |
| Desire for simple solutions | 2000 | 3.25 | 0.96 | 1 | 5 |
| Scientific Literacy | 1993 | 3.98 | 1.24 | 0 | 6 |
| Confidence in Scientific Community | 1999 | 3.81 | 1.02 | 1 | 5 |
| Anti-intellectualism | 2000 | 3.09 | 1.15 | 1 | 7 |
| Conspiracy thinking | 1999 | 3.15 | 1.04 | 1 | 5 |
| Positive Affect | 1995 | 30.54 | 8.65 | 10 | 50 |
| Negative Affect | 1998 | 20.02 | 8.79 | 10 | 50 |
| Legacy Mainstream News Media Use | 2000 | 3.19 | 1.13 | 1 | 5 |
| Online Mainstream News Media Use | 2001 | 2.71 | 1.28 | 1 | 5 |
| Mainstream Social Media Use | 2001 | 2.93 | 1.33 | 1 | 5 |
| Alternative Social Media Use | 2001 | 1.72 | 1.02 | 1 | 5 |

**Main Dependent variable of interest**

**Independent variables**

**Psychological factors: Personality characteristics and thinking styles**

Need for Chaos^[[1]](#endnote-1)^

Here are some ideas that some people agree with, and others disagree with. In thinking about each one, please tell us how much you agree or disagree with each of the statements below: (percent agree/strongly agree in parentheses)

- I fantasize about a natural disaster wiping out most of humanity such that a small group of people can start all over. (17.37)
- I think society should be burned to the ground. (14.46)
- When I think about our political institutions, I cannot help thinking "just let them all burn." (21.00)
- We cannot fix the problems in our society, we need to tear it down and start over. (20.57)
- I need chaos around me – it is too boring if nothing is going on. (15.22)
- Sometimes I just feel like destroying beautiful things. (13.60)

Dark Tetrad^[[2]](#endnote-2)^

*Machiavellianism*

Please tell us how much you agree or disagree with each of the statements below: (percent agree/strongly agree in parentheses)

- I tend to manipulate others to get my way. (15.34)
- I have used deceit or lied to get my way. (22.20)
- I have used flattery to get my way. (31.43)
- I tend to exploit others towards my own end. (14.34)

*Narcissism*

Please tell us how much you agree or disagree with each of the statements below: (percent agree/strongly agree in parentheses)

- I tend to want others to admire me. (30.22)
- I tend to want others to pay attention to me. (30.07)
- I tend to seek prestige or status. (23.57)
- I tend to expect special favors from others. (19.26)

*Psychopathy*

Please tell us how much you agree or disagree with each of the statements below: (percent agree/strongly agree in parentheses)

- I tend to lack remorse. (14.55)
- I tend to be unconcerned with the morality of my actions. (15.80)
- I tend to be callous or insensitive. (16.25)
- I tend to be cynical. (26.00)

*Sadism*^[[3]](#endnote-3)^

Please tell us how much you agree or disagree with each of the statements below: (percent agree/strongly agree in parentheses)

- It's funny when idiots fall flat on their face. (34.92)
- Some people deserve to suffer. (26.66)
- Just for kicks, I've said mean things on social media. (18.05)
- I know how to hurt someone with words alone. (46.95)

Paranoia^[[4]](#endnote-4)^

Please tell us how much you agree or disagree with each of the statements below: (percent agree/strongly agree in parentheses)

- Certain people have it in for me. (20.57)
- There is a conspiracy against me. (13.45)
- People are persecuting me. (13.90)

Dogmatism^[[5]](#endnote-5)^

Please tell us how much you agree or disagree with each of the statements below: (percent agree/strongly agree in parentheses)

- On important public issues, I believe you should always be uncompromising, and either be for them or against them. (33.20)
- It is better to take a stand on an issue even if it's wrong. (32.85)
- When it comes to the really important questions about religion and philosophy of life, a person must decide them, one way or the other. (51.50)

Conspiracy Thinking^[[6]](#endnote-6)^

Here are some ideas that some people agree with, and others disagree with. In thinking about each one, please tell us how much you agree or disagree with each of the statements below: (percent agree/strongly agree in parentheses)

- Even though we live in a democracy, a few people will always run things anyway. (59.28)
- The people who really “run” the country, are not known to the voters. (44.19)
- Big events like wars, the recent recession, and the outcomes of elections are controlled by small groups of people who are working in secret against the rest of us. (35.64)
- Much of our lives are being controlled by plots hatched in secret places. (34.27)

Right-Wing Authoritarianism^[[7]](#endnote-7)^

Please tell us how much you agree or disagree with each of the statements below:

- God's laws about abortion, pornography, and marriage must be strictly followed before it is too late. (percent agree/strongly agree: 40.60)
- There is nothing wrong with premarital sexual intercourse. (percent disagree/strongly disagree: 20.85)
- Our society needs stricter laws. (percent agree/strongly agree: 47.75)
- Crime and the recent public disorders show that we have to crack down harder on troublemakers, if we are going preserve law and order. (percent agree/strongly agree: 63.40)

Left-Wing Authoritarianism^[[8]](#endnote-8)^

Please tell us how much you agree or disagree with each of the statements below: (percent agree/strongly agree in parentheses)

- The rich should be stripped of their belongings and status. (19.40)
- Rich people should be forced to give up most of their wealth. (23.99)
- If I could remake society, I would put people who currently have the most privilege at the very bottom. (27.09)

Victimhood^[[9]](#endnote-9)^

Please tell us how much you agree or disagree with each of the statements below: (percent agree/strongly agree in parentheses)

- I rarely get what I deserve in life. (26.85)
- Great things never come to me. (25.36)
- I usually have to settle for less. (34.72)
- I never seem to get an extra break. (32.55)

General Conflict Tactic Scale^[[10]](#endnote-10)^

During the past 12 months, have you done the following things when having a disagreement with another person? Yes = 1, No = 0 (scores can range from 0 to 6)

Insulted or swore at someone?

Pushed, grabbed, or shoved someone?

Threatened to hit another person?

Hit, kicked, bit, or slapped someone?

Beat up someone?

Threatened to use or actually used a knife or gun on someone?

Argumentativeness^[[11]](#endnote-11)^

Here are some ideas that some people agree with, and others disagree with. In thinking about each one, please tell us how much you agree or disagree with each of the statements below:

I like to argue online with other people.

I enjoy a good argument over a controversial issue.

I am willing to express my opinion online even if others strongly disagree with me.

Intolerance of Uncertainty^[[12]](#endnote-12)^

(rated “strongly agree” to “strongly disagree.” Reverse-scored for analysis)

Uncertainty keeps me from living a full life.

When I am uncertain, I can’t function very well.

I must get away from uncertain situations.

Subjective Numeracy Scale (short form: SNS3)^[[13]](#endnote-13)^

(Rated from 1 = Not at all good or never to 6 = Extremely good or very often)

How good are you at figuring out how much a shirt will cost if it is 25% off?

How good are you at working with fractions?

How often do you find numerical information to be useful?

**Psychological factors: Media use**

Perceived sense of power/control^[[14]](#endnote-14)^

(rated “strongly agree” to “strongly disagree.” Reverse-scored for analysis. Only those who answered “Yes” to the question “Do you ever post on social media?” were shown this question.)

When posting on social media, I have a great deal of power.

I am able to get my way when expressing my opinions on social media.

I can get people commenting on my social media posts to say what I want.

Social media self-expression^[[15]](#endnote-15)^

(rated “strongly agree” to “strongly disagree.” Reverse-scored for analysis)

When on the internet or social media, I generally express the aspects of myself that I don’t feel comfortable expressing in person.

I frequently and generally use the internet and social media to express “who I really am.”

I generally do and say things online that I wouldn’t say or do in person.

**Psychological factors: Affect/emotion**

Positive and Negative Affect Schedule^[[16]](#endnote-16)^

The twenty emotions in this schedule were broken up into four groups of five. The order of the blocks, and the order of the emotions listed in them, were randomized. For each emotion the response options are: “very slightly or not at all,” “a little,” “moderately,” “quite a bit,” “extremely.”

Indicate the extent you have felt this way over the past week. (percent quite a bit/extremely in parentheses)

- Interested (45.70)
- Distressed (22.30)
- Excited (31.03)
- Upset (22.21)
- Strong (38.07)

Indicate the extent you have felt this way over the past week.

- Guilty (8.91)
- Scared (12.01)
- Hostile (10.26)
- Enthusiastic (32.02)
- Proud (33.47)

Indicate the extent you have felt this way over the past week.

- Irritable (19.97)
- Alert (46.37)
- Ashamed (13.77)
- Inspired (33.42)
- Nervous (20.62)

Indicate the extent you have felt this way over the past week.

- Determined (45.20)
- Attentive (46.47)
- Jittery (17.35)
- Active (41.35)
- Afraid (15.95)

**Sociological Factors: Religious**

Religiosity

*Importance of Religion in One’s Life*

How important is religion in your life? (percentages in parentheses)

- Very important (40.05)
- Somewhat important (27.50)
- Not too important (13.85)
- Not at all important (18.60)

Variable reverse coded for analysis.

*Frequency of Service Attendance*

Aside from weddings and funerals, how often do you attend religious services? (percentages in parentheses)

- More than once a week (10.22)
- Once a week (19.06)
- Once or twice a month (10.12)
- A few times a year (14.49)
- Seldom (17.44)
- Never (28.67)
- Don't know (0.00)

Variable reverse coded for analysis. There were no “don’t know” responses.

*Frequency of Prayer*

People practice their religion in different ways. Outside of attending religious services, how often do you pray? (percentages in parentheses)

- Several times a day (28.40)
- Once a day (15.80)
- A few times a week (15.05)
- Once a week (3.80)
- A few times a month (6.35)
- Seldom (12.75)
- Never (16.05)
- Don't know (1.80)

Variable reverse coded for analysis. “Never” (n = 321) and “don’t know” (n = 36) responses were combined.

**Political Factors**

Partisanship

Generally speaking, do you usually think of yourself as a Republican, a Democrat, an Independent, or something else? (percentages in parentheses)

- Democrat (44.18)
- Republican (26.39)
- Independent (24.54)
- Something else (4.90)

[For respondents answering “Democrat” or “Republican”] Would you call yourself a strong [Democrat/Republican] or a not very strong [Democrat/Republican]? (percentages in parentheses)

- Strong (Democrats: 68.10; Republicans: 62.31)
- Not very strong (Democrats: 31.90; Republicans: 37.69)

[For respondents answering “Independent or “Something else”] Do you think of yourself as closer to the Republican or Democratic Party? (percentages in parentheses)

- Republican (18.68)
- Democrat (24.79)
- Neither (56.54)

Ideology

Where would you place yourself on a scale that goes from “very liberal” to “very conservative”? (percentages in parentheses)

- Very liberal (14.45)
- Liberal (12.20)
- Slightly liberal (7.55)
- Moderate (36.10)
- Slightly conservative (7.20)
- Conservative (11.15)
- Very conservative (11.35)

**Information Environment**

Legacy News Media Use

How often do you use the following media to get information about current events, public issues, or politics? (percent everyday/several times a week in parentheses)

- Network TV news (58.60)
- Cable TV news (54.10)
- Local TV news (67.20)
- Print newspapers (34.15)
- Radio (49.45)

The presentation order of these news outlets was randomized. Response options: “Every day,” “Several times a week,” “Several times a month,” “Once a month or less,” “Never.”

Online News Media Use

How often do you use the following media to get information about current events, public issues, or politics? (percent everyday/several times a week in parentheses)

- Online newspapers (38.90)
- Online news magazines (29.82)
- Blogs (19.95)
- YouTube (54.17)
- Facebook (58.95)
- Twitter (32.15)
- Reddit (20.44)
- Instagram (38.50)
- TV news websites (e.g., CNN.com, FoxNews.com) (51.43)
- 8Kun (7.20)
- Telegram (17.00)
- Truth Social (14.25)

The presentation order of these news outlets was randomized. Response options (reverse coded for analysis): “Every day,” “Several times a week,” “Several times a month,” “Once a month or less,” “Never.”

Follows Politics

Please tell us how much you agree or disagree with each of the statements below: (percent agree/strongly agree in parentheses)

- I closely follow what's going on in government and current events. (62.80)

**Attitudes Towards Science, Experts, and Authority**

Scientific Literacy^[[17]](#endnote-17)^

To the best of your knowledge, are the following statements true or false? (percent correct response in parentheses)

- The center of the Earth is very cold. (79.84)
- The oxygen we breathe comes from plants. (85.39)
- Atoms are smaller than electrons. (47.52)
- The earliest humans lived at the same time as the dinosaurs. (59.03)
- Human beings, as we know them today, developed from earlier species of animals. (59.56)
- It is the father’s gene that decides whether the baby is a boy or a girl. (67.18)

Confidence in the Scientific Community

I have confidence in the scientific community. (percentages in parentheses)

- Strongly agree (27.31)
- Agree (39.82)
- Neither agree nor disagree (22.76)
- Disagree (6.85)
- Strongly disagree (3.25)

**Other political orientations and traits**

Christian Nationalism

(rated “strongly agree” to “strongly disagree.”)

- The federal government should declare the United States a Christian nation.
- The federal government should advocate Christian values.
- The federal government should enforce strict separation of church and state (reverse coded).
- The success of the United States is part of God’s plan.
- The federal government should allow prayer in public schools.

Denialism

(rated “strongly agree” to “strongly disagree.”)

- Much of the information we receive is wrong.
- I often disagree with conventional views about the world.
- Official government accounts of events cannot be trusted.
- Major events are not always what they seem.
- Much of the mainstream news is deliberately slanted to mislead us.

National Narcissism

(rated “strongly agree” to “strongly disagree.”)

- The United States deserves special treatment.
- Not many people seem to fully understand the importance of the United States.
- I will never be satisfied until the United States gets the recognition it deserves.

Populism

(rated “strongly agree” to “strongly disagree.”)

- Elected officials talk too much and take too little action.
- What people call "compromise" in politics is really just selling out on one's principles.
- Established politicians who claim to defend our interests only take care of themselves
- The established elite and politicians have often betrayed the people.
- Politics is a battle between good and evil.

**A3. Finding #2: Correlations with demographic variables**

**Table A3. Point-biserial correlations with demographic variables**

| Variables | (1) | (2) | (3) | (4) | (5) | (6) | (7) | (8) | (9) |
| --- | --- | --- | --- | --- | --- | --- | --- | --- | --- |
| (1) Cryptocurrency | 1.000 |  |  |  |  |  |  |  |  |
|  |  |  |  |  |  |  |  |  |  |
| (2) Sex (female) | -0.244^***^ | 1.000 |  |  |  |  |  |  |  |
|  | (0.000) |  |  |  |  |  |  |  |  |
| (3) Age | -0.358^***^ | -0.058^***^ | 1.000 |  |  |  |  |  |  |
|  | (0.000) | (0.010) |  |  |  |  |  |  |  |
| (4) Income | 0.202^***^ | -0.353^***^ | 0.156^***^ | 1.000 |  |  |  |  |  |
|  | (0.000) | (0.000) | (0.000) |  |  |  |  |  |  |
| (5) Education | 0.203^***^ | -0.236^***^ | 0.096^***^ | 0.555^***^ | 1.000 |  |  |  |  |
|  | (0.000) | (0.000) | (0.000) | (0.000) |  |  |  |  |  |
| (6) Religiosity | 0.200^***^ | 0.007 | -0.054^*^ | 0.055^*^ | 0.070^**^ | 1.000 |  |  |  |
|  | (0.000) | (0.769) | (0.016) | (0.014) | (0.002) |  |  |  |  |
| (7) White | -0.041 | -0.049^*^ | 0.298^***^ | 0.161^***^ | 0.137^***^ | -0.045^*^ | 1.000 |  |  |
|  | (0.065) | (0.027) | (0.000) | (0.000) | (0.000) | (0.044) |  |  |  |
| (8) Black | 0.066^**^ | 0.073^***^ | -0.285^***^ | -0.182^***^ | -0.150^***^ | 0.094^***^ | -0.751^***^ | 1.000 |  |
|  | (0.003) | (0.001) | (0.000) | (0.000) | (0.000) | (0.000) | (0.000) |  |  |
| (9) Hispanic | 0.073^***^ | 0.065^**^ | -0.304^***^ | -0.067^**^ | -0.041 | 0.019 | -0.152^***^ | -0.004 | 1.000 |
|  | (0.001) | (0.004) | (0.000) | (0.003) | (0.066) | (0.404) | (0.000) | (0.851) |  |
| **** p<0.001, ** p<0.01, * p<0.05* | | | | | | | | | |

**A4. Finding #3: Thinking styles and attitudes toward science**

**Table A4. Point-biserial correlations with measures of thinking styles and attitudes toward science**

| Variables | (1) | (2) | (3) | (4) | (5) | (6) | (7) | (8) | (9) |
| --- | --- | --- | --- | --- | --- | --- | --- | --- | --- |
| (1) Cryptocurrency | 1.000 |  |  |  |  |  |  |  |  |
|  |  |  |  |  |  |  |  |  |  |
| (2) Patternicity | 0.303^***^ | 1.000 |  |  |  |  |  |  |  |
|  | (0.000) |  |  |  |  |  |  |  |  |
| (3) Subjective numeracy | 0.181^***^ | 0.352^***^ | 1.000 |  |  |  |  |  |  |
|  | (0.000) | (0.000) |  |  |  |  |  |  |  |
| (4) Desire for simple solutions | 0.274^***^ | 0.319^***^ | 0.088^***^ | 1.000 |  |  |  |  |  |
|  | (0.000) | (0.000) | (0.000) |  |  |  |  |  |  |
| (5) Intolerance of uncertainty | 0.177^***^ | 0.271^***^ | -0.056^*^ | 0.323^***^ | 1.000 |  |  |  |  |
|  | (0.000) | (0.000) | (0.012) | (0.000) |  |  |  |  |  |
| (6) Science literacy | 0.122^***^ | 0.144^***^ | -0.034 | 0.229^***^ | 0.206^***^ | 1.000 |  |  |  |
|  | (0.000) | (0.000) | (0.124) | (0.000) | (0.000) |  |  |  |  |
| (7) Conspiracy thinking | 0.229^***^ | 0.291^***^ | -0.009 | 0.428^***^ | 0.327^***^ | 0.193^***^ | 1.000 |  |  |
|  | (0.000) | (0.000) | (0.675) | (0.000) | (0.000) | (0.000) |  |  |  |
| (8) Total conspiracy theories | 0.325^***^ | 0.354^***^ | 0.092^***^ | 0.451^***^ | 0.355^***^ | 0.258^***^ | 0.663^***^ | 1.000 |  |
| believed | (0.000) | (0.000) | (0.000) | (0.000) | (0.000) | (0.000) | (0.000) |  |  |
| (9) Anti-intellectualism | -0.162^***^ | -0.158^***^ | -0.219^***^ | -0.121^***^ | -0.068^**^ | -0.125^***^ | 0.195^***^ | 0.108^***^ | 1.000 |
|  | (0.000) | (0.000) | (0.000) | (0.000) | (0.002) | (0.000) | (0.000) | (0.000) |  |
| (10) Confidence in scientific | 0.150^***^ | 0.163^***^ | 0.244^***^ | 0.024 | 0.045^*^ | 0.059^**^ | -0.179^***^ | -0.126^***^ | -0.580^***^ |
| community | (0.000) | (0.000) | (0.000) | (0.292) | (0.042) | (0.008) | (0.000) | (0.000) | (0.000) |

**** p<0.001, ** p<0.01, * p<0.05*

**A5. Finding #4: Left-right political orientations**

**Table A4. Point-biserial correlations with partisanship and ratings of political figures and groups**

| Variables | (1) | (2) | (3) | (4) | (5) | (6) | (7) | (8) | (9) |
| --- | --- | --- | --- | --- | --- | --- | --- | --- | --- |
| (1) Cryptocurrency | 1.000 |  |  |  |  |  |  |  |  |
|  |  |  |  |  |  |  |  |  |  |
| (2) Democrat Party | 0.172^***^ | 1.000 |  |  |  |  |  |  |  |
|  | (0.000) |  |  |  |  |  |  |  |  |
| (3) Rep. Party | 0.088^***^ | -0.283^***^ | 1.000 |  |  |  |  |  |  |
|  | (0.000) | (0.000) |  |  |  |  |  |  |  |
| (4) Joe Biden | 0.173^***^ | 0.856^***^ | -0.310^***^ | 1.000 |  |  |  |  |  |
|  | (0.000) | (0.000) | (0.000) |  |  |  |  |  |  |
| (5) Bernie Sanders | 0.147^***^ | 0.672^***^ | -0.233^***^ | 0.643^***^ | 1.000 |  |  |  |  |
|  | (0.000) | (0.000) | (0.000) | (0.000) |  |  |  |  |  |
| (6) Donald Trump | 0.109^***^ | -0.349^***^ | 0.789* | -0.405^***^ | -0.284^***^ | 1.000 |  |  |  |
|  | (0.000) | (0.000) | (0.000) | (0.000) | (0.000) |  |  |  |  |
| (7) Progressives | 0.234^***^ | 0.518^***^ | -0.006 | 0.503^***^ | 0.519^***^ | -0.048^*^ | 1.000 |  |  |
|  | (0.000) | (0.000) | (0.777) | (0.000) | (0.000) | (0.035) |  |  |  |
| (8) Party (Dem-Rep) | -0.166^***^ | -0.690^***^ | 0.559^***^ | -0.655^***^ | -0.515^***^ | 0.539^***^ | -0.344^***^ | 1.000 |  |
|  | (0.000) | (0.000) | (0.000) | (0.000) | (0.000) | (0.000) | (0.000) |  |  |
| (9) Ideology (Lib-Con) | -0.095^***^ | -0.451^***^ | 0.381^***^ | -0.433^***^ | -0.455^***^ | 0.380^***^ | -0.351^***^ | 0.563^***^ | 1.000 |
|  | (0.000) | (0.000) | (0.000) | (0.000) | (0.000) | (0.000) | (0.000) | (0.000) |  |
| **** p<0.001, * p<0.05* | | | | | | | | | |

**A6. Finding #5: Non-Left/Right Political Orientations**

**Table A5. Point-biserial correlations with measures of non-left/right Political Orientations**

| Variables | (1) | (2) | (3) | (4) | (5) | (6) | (7) | (8) | (9) | (10) |
| --- | --- | --- | --- | --- | --- | --- | --- | --- | --- | --- |
| (1) Cryptocurrency | 1.000 |  |  |  |  |  |  |  |  |  |
|  |  |  |  |  |  |  |  |  |  |  |
| (2) Political interest | 0.173^***^ | 1.000 |  |  |  |  |  |  |  |  |
|  | (0.000) |  |  |  |  |  |  |  |  |  |
| (3) Political efficacy | 0.213^***^ | 0.363^***^ | 1.000 |  |  |  |  |  |  |  |
|  | (0.000) | (0.000) |  |  |  |  |  |  |  |  |
| (4) Populism | 0.106^***^ | 0.083^***^ | 0.012 | 1.000 |  |  |  |  |  |  |
|  | (0.000) | (0.000) | (0.594) |  |  |  |  |  |  |  |
| (5) Trust in people | 0.104^***^ | 0.227^***^ | 0.308^***^ | -0.076^**^ | 1.000 |  |  |  |  |  |
|  | (0.000) | (0.000) | (0.000) | (0.001) |  |  |  |  |  |  |
| (6) Trust in government | 0.178^***^ | 0.189^***^ | 0.376^***^ | -0.225^***^ | 0.528^***^ | 1.000 |  |  |  |  |
|  | (0.000) | (0.000) | (0.000) | (0.000) | (0.000) |  |  |  |  |  |
| (7) Trust in police | 0.034 | 0.184^***^ | 0.210^***^ | -0.051^*^ | 0.514^***^ | 0.485^***^ | 1.000 |  |  |  |
|  | (0.124) | (0.000) | (0.000) | (0.023) | (0.000) | (0.000) |  |  |  |  |
| (8) Denialism | 0.102^***^ | 0.012 | -0.022 | 0.497^***^ | -0.145^***^ | -0.296^***^ | -0.103^***^ | 1.000 |  |  |
|  | (0.000) | (0.603) | (0.322) | (0.000) | (0.000) | (0.000) | (0.000) |  |  |  |
| (9) Christian Nationalism | 0.155^***^ | 0.066^***^ | 0.154^***^ | 0.267^***^ | 0.092^***^ | 0.068^**^ | 0.171^***^ | 0.299^***^ | 1.000 |  |
|  | (0.000) | (0.003) | (0.000) | (0.000) | (0.000) | (0.002) | (0.000) | (0.000) |  |  |
| (10) National narcissism | 0.200^***^ | 0.154^***^ | 0.217^***^ | 0.254^***^ | 0.147^***^ | 0.168^***^ | 0.238^***^ | 0.250^***^ | 0.503^***^ | 1.000 |
|  | (0.000) | (0.000) | (0.000) | (0.000) | (0.000) | (0.000) | (0.000) | (0.000) | (0.000) |  |
| (11) Gendered nationalism | 0.211^***^ | 0.109^***^ | 0.120^***^ | 0.325^***^ | 0.055^*^ | 0.017 | 0.178^***^ | 0.408^***^ | 0.580^***^ | 0.539^***^ |
|  | (0.000) | (0.000) | (0.000) | (0.000) | (0.014) | (0.449) | (0.000) | (0.000) | (0.000) | (0.000) |

**** p<0.001, ** p<0.01, * p<0.05*

**A7. Finding #6: Left/right political extremity**

**Table A6. Point-biserial correlations with measures of left/right political extremity**

| Variables | (1) | (2) | (3) | (4) | (5) | (6) | (7) | (8) | (9) |
| --- | --- | --- | --- | --- | --- | --- | --- | --- | --- |
| (1) Cryptocurrency | 1.000 |  |  |  |  |  |  |  |  |
|  |  |  |  |  |  |  |  |  |  |
| (2) Partisan intensity | 0.156^***^ | 1.000 |  |  |  |  |  |  |  |
|  | (0.000) |  |  |  |  |  |  |  |  |
| (3) Ideological intensity | 0.148^***^ | 0.420^***^ | 1.000 |  |  |  |  |  |  |
|  | (0.000) | (0.000) |  |  |  |  |  |  |  |
| (4) Vladimir Putin | 0.340^***^ | 0.104^***^ | 0.081^***^ | 1.000 |  |  |  |  |  |
|  | (0.000) | (0.000) | (0.000) |  |  |  |  |  |  |
| (5) QAnon | 0.347^***^ | 0.178^***^ | 0.157^***^ | 0.657^***^ | 1.000 |  |  |  |  |
|  | (0.000) | (0.000) | (0.000) | (0.000) |  |  |  |  |  |
| (6) Proud Boys | 0.325^***^ | 0.172^***^ | 0.169^***^ | 0.641^***^ | 0.778^***^ | 1.000 |  |  |  |
|  | (0.000) | (0.000) | (0.000) | (0.000) | (0.000) |  |  |  |  |
| (7) White Nationalists | 0.350^***^ | 0.201^***^ | 0.147^***^ | 0.593^***^ | 0.713^***^ | 0.759^***^ | 1.000 |  |  |
|  | (0.000) | (0.000) | (0.000) | (0.000) | (0.000) | (0.000) |  |  |  |
| (8) Antifa | 0.278^***^ | 0.159^***^ | 0.177^***^ | 0.559^***^ | 0.643^***^ | 0.598^***^ | 0.563^***^ | 1.000 |  |
|  | (0.000) | (0.000) | (0.000) | (0.000) | (0.000) | (0.000) | (0.000) |  |  |
| (9) LW authoritarianism | 0.259^***^ | 0.081^***^ | 0.075^**^ | 0.316^***^ | 0.348^***^ | 0.319^***^ | 0.320^***^ | 0.356^***^ | 1.000 |
|  | (0.000) | (0.000) | (0.001) | (0.000) | (0.000) | (0.000) | (0.000) | (0.000) |  |
| (10) RW authoritarianism | 0.091^***^ | 0.162^***^ | 0.058^*^ | 0.234^***^ | 0.272^***^ | 0.303^***^ | 0.332^***^ | 0.059^*^ | 0.153^***^ |
|  | (0.000) | (0.000) | (0.010) | (0.000) | (0.000) | (0.000) | (0.000) | (0.010) | (0.000) |

**** p<0.001, ** p<0.01, * p<0.05*

**A8. Finding #7: Interpersonal and political behaviors**

**Table A7. Point-biserial correlations with measures of interpersonal and political behaviors**

| Variables | (1) | (2) | (3) | (4) | (5) | (6) | (7) | (8) | (9) | (10) |
| --- | --- | --- | --- | --- | --- | --- | --- | --- | --- | --- |
| (1) Cryptocurrency | 1.000 |  |  |  |  |  |  |  |  |  |
|  |  |  |  |  |  |  |  |  |  |  |
| (2) Political protests | 0.342^***^ | 1.000 |  |  |  |  |  |  |  |  |
|  | (0.000) |  |  |  |  |  |  |  |  |  |
| (3) Political meetings | 0.371^***^ | 0.655^***^ | 1.000 |  |  |  |  |  |  |  |
|  | (0.000) | (0.000) |  |  |  |  |  |  |  |  |
| (4) Contacted official | 0.225^***^ | 0.512^***^ | 0.557^***^ | 1.000 |  |  |  |  |  |  |
|  | (0.000) | (0.000) | (0.000) |  |  |  |  |  |  |  |
| (5) Political volunteer | 0.373^***^ | 0.611^***^ | 0.704^***^ | 0.536^***^ | 1.000 |  |  |  |  |  |
|  | (0.000) | (0.000) | (0.000) | (0.000) |  |  |  |  |  |  |
| (6) Civil disobedience | 0.326^***^ | 0.669^***^ | 0.616^***^ | 0.507^***^ | 0.613^***^ | 1.000 |  |  |  |  |
|  | (0.000) | (0.000) | (0.000) | (0.000) | (0.000) |  |  |  |  |  |
| (7) Political violence | 0.332^***^ | 0.660^***^ | 0.607^***^ | 0.490^***^ | 0.616^***^ | 0.763^***^ | 1.000 |  |  |  |
|  | (0.000) | (0.000) | (0.000) | (0.000) | (0.000) | (0.000) |  |  |  |  |
| (8) Argumentativeness | 0.383^***^ | 0.401^***^ | 0.381^***^ | 0.284^***^ | 0.378^***^ | 0.368^***^ | 0.353^***^ | 1.000 |  |  |
|  | (0.000) | (0.000) | (0.000) | (0.000) | (0.000) | (0.000) | (0.000) |  |  |  |
| (9) Conflict escalation | 0.269^***^ | 0.388^***^ | 0.324^***^ | 0.230^***^ | 0.330^***^ | 0.432^***^ | 0.424^***^ | 0.343^***^ | 1.000 |  |
|  | (0.000) | (0.000) | (0.000) | (0.000) | (0.000) | (0.000) | (0.000) | (0.000) |  |  |
| (10) Qualified for office | 0.333^***^ | 0.379^***^ | 0.423^***^ | 0.343^***^ | 0.418^***^ | 0.356^***^ | 0.371^***^ | 0.461^***^ | 0.255^***^ | 1.000 |
|  | (0.000) | (0.000) | (0.000) | (0.000) | (0.000) | (0.000) | (0.000) | (0.000) | (0.000) |  |
| (11) Might run for office | 0.416^***^ | 0.477^***^ | 0.504^***^ | 0.334^***^ | 0.502^***^ | 0.471^***^ | 0.484^***^ | 0.491^***^ | 0.359^***^ | 0.725^***^ |
|  | (0.000) | (0.000) | (0.000) | (0.000) | (0.000) | (0.000) | (0.000) | (0.000) | (0.000) | (0.000) |

**** p<0.001*

**A9. Finding #8: Personality, emotional, and motivational characteristics**

**Table A8. Point-biserial correlations with measures of personality, emotional, and motivational characteristics**

| Variables | (1) | (2) | (3) | (4) | (5) | (6) | (7) | (8) | (9) | | (10) | | (11) | | (12) | | (13) | | (14) | | (15) | | |
| --- | --- | --- | --- | --- | --- | --- | --- | --- | --- | --- | --- | --- | --- | --- | --- | --- | --- | --- | --- | --- | --- | --- | --- |
| (1) Cryptocurrency | 1.000 |  |  |  |  |  |  |  |  | |  | |  | |  | |  | |  | |  | | |
|  |  |  |  |  |  |  |  |  |  | |  | |  | |  | |  | |  | |  | | |
| (2) Need for Chaos | 0.350^***^ | 1.000 |  |  |  |  |  |  |  | |  | |  | |  | |  | |  | |  | | |
|  | (0.000) |  |  |  |  |  |  |  |  | |  | |  | |  | |  | |  | |  | | |
| (3) Reactance | 0.111^***^ | 0.281^***^ | 1.000 |  |  |  |  |  |  | |  | |  | |  | |  | |  | |  | | |
|  | (0.000) | (0.000) |  |  |  |  |  |  |  | |  | |  | |  | |  | |  | |  | | |
| (4) Narcissism | 0.382^***^ | 0.517^***^ | 0.207^***^ | 1.000 |  |  |  |  |  | |  | |  | |  | |  | |  | |  | | |
|  | (0.000) | (0.000) | (0.000) |  |  |  |  |  |  | |  | |  | |  | |  | |  | |  | | |
| (5) Psychopathy | 0.271^***^ | 0.626^***^ | 0.299^***^ | 0.483^***^ | 1.000 |  |  |  |  | |  | |  | |  | |  | |  | |  | | |
|  | (0.000) | (0.000) | (0.000) | (0.000) |  |  |  |  |  | |  | |  | |  | |  | |  | |  | | |
| (6) Machiavellianism | 0.288^***^ | 0.594^***^ | 0.275^***^ | 0.533^***^ | 0.624^***^ | 1.000 |  |  |  | |  | |  | |  | |  | |  | |  | | |
|  | (0.000) | (0.000) | (0.000) | (0.000) | (0.000) |  |  |  |  | |  | |  | |  | |  | |  | |  | | |
| (7) Sadism | 0.273^***^ | 0.561^***^ | 0.333^***^ | 0.416^***^ | 0.546^***^ | 0.561^***^ | 1.000 |  |  | |  | |  | |  | |  | |  | |  | | |
|  | (0.000) | (0.000) | (0.000) | (0.000) | (0.000) | (0.000) |  |  |  | |  | |  | |  | |  | |  | |  | | |
| (8) Victimhood | 0.176^***^ | 0.453^***^ | 0.294^***^ | 0.243^***^ | 0.396^***^ | 0.352^***^ | 0.374^***^ | 1.000 |  |  | |  | |  | |  | |  | |  | | |  |
|  | (0.000) | (0.000) | (0.000) | (0.000) | (0.000) | (0.000) | (0.000) |  |  | |  | |  | |  | |  | |  | |  | | |
| (9) Dogmatism | 0.232^***^ | 0.411^***^ | 0.288^***^ | 0.358^***^ | 0.375^***^ | 0.293^***^ | 0.319^***^ | 0.247^***^ | 1.000 | |  | |  | |  | |  | |  | |  | | |
|  | (0.000) | (0.000) | (0.000) | (0.000) | (0.000) | (0.000) | (0.000) | (0.000) |  | |  | |  | |  | |  | |  | |  | | |
| (10) Impulsiveness | -0.083^***^ | 0.101^***^ | 0.045^***^ | -0.062^***^ | 0.121^***^ | 0.121^***^ | 0.069^**^ | 0.189^***^ | -0.095^***^ | | 1.000 | |  | |  | |  | |  | |  | | |
|  | (0.000) | (0.000) | (0.043) | (0.005) | (0.000) | (0.000) | (0.002) | (0.000) | (0.000) | |  | |  | |  | |  | |  | |  | | |
| (11) Schizotypy | 0.354^***^ | 0.585^***^ | 0.326^***^ | 0.530^***^ | 0.475^***^ | 0.478^***^ | 0.464^***^ | 0.405^***^ | 0.445^***^ | | 0.017 | | 1.000 | |  | |  | |  | |  | | |
|  | (0.000) | (0.000) | (0.000) | (0.000) | (0.000) | (0.000) | (0.000) | (0.000) | (0.000) | | (0.439) | |  | |  | |  | |  | |  | | |
| (12) Paranoia | 0.351^***^ | 0.652^***^ | 0.299^***^ | 0.530^***^ | 0.592^***^ | 0.546^***^ | 0.523^***^ | 0.466^***^ | 0.425^***^ | | 0.062^**^ | | 0.609^***^ | | 1.000 | |  | |  | |  | | |
|  | (0.000) | (0.000) | (0.000) | (0.000) | (0.000) | (0.000) | (0.000) | (0.000) | (0.000) | | (0.005) | | (0.000) | |  | |  | |  | |  | | |
| (13) Agentic goals | 0.312^***^ | 0.327^***^ | 0.152^***^ | 0.540^***^ | 0.278^***^ | 0.312^***^ | 0.261^***^ | 0.152^***^ | 0.320^***^ | | -0.186^***^ | | 0.429^***^ | | 0.373^***^ | | 1.000 | |  | |  | | |
|  | (0.000) | (0.000) | (0.000) | (0.000) | (0.000) | (0.000) | (0.000) | (0.000) | (0.000) | | (0.000) | | (0.000) | | (0.000) | |  | |  | |  | | |
| (14) Communal goals | 0.080^***^ | -0.072^**^ | 0.045^*^ | 0.090^***^ | -0.179^***^ | -0.088^***^ | -0.090^***^ | -0.085^***^ | 0.110^***^ | | -0.239^***^ | | 0.117^***^ | | -0.011 | | 0.392^***^ | | 1.000 | |  | | |
|  | (0.000) | (0.001) | (0.042) | (0.000) | (0.000) | (0.000) | (0.000) | (0.000) | (0.000) | | (0.000) | | (0.000) | | (0.631) | | (0.000) | |  | |  |  |  |
| (15) Positive affect | 0.163^***^ | 0.047^*^ | 0.012 | 0.198^***^ | -0.002 | 0.017 | 0.051^*^ | -0.202^***^ | 0.167^***^ | | -0.381^***^ | | 0.137^***^ | | 0.072^**^ | | 0.352^***^ | | 0.322^***^ | | 1.000 | | |
|  | (0.000) | (0.036) | (0.591) | (0.000) | (0.915) | (0.451) | (0.022) | (0.000) | (0.000) | | (0.000) | | (0.000) | | (0.001) | | (0.000) | | (0.000) | |  | | |
| (16) Negative affect | 0.247^***^ | 0.481^***^ | 0.261^***^ | 0.378^***^ | 0.410^***^ | 0.410^***^ | 0.361^***^ | 0.468^***^ | 0.258^***^ | | 0.183^***^ | | 0.460^***^ | | 0.497^***^ | | 0.267^***^ | | 0.012 | | -0.054^*^ | | |
|  | (0.000) | (0.000) | (0.000) | (0.000) | (0.000) | (0.000) | (0.000) | (0.000) | (0.000) | | (0.000) | | (0.000) | | (0.000) | | (0.000) | | (0.577) | | (0.016) | | |

**** p<0.001, ** p<0.01, * p<0.05*

**A10. Finding #9: Informational environment and media use**

**Table A9a. Point-biserial correlations with frequency of media use (by category)**

| Variables | (1) | (2) | (3) | (4) |
| --- | --- | --- | --- | --- |
| (1) Cryptocurrency | 1.000 |  |  |  |
|  |  |  |  |  |
| (2) Legacy media (offline) | 0.234^***^ | 1.000 |  |  |
|  | (0.000) |  |  |  |
| (3) Legacy media (online) | 0.341^***^ | 0.610^***^ | 1.000 |  |
|  | (0.000) | (0.000) |  |  |
| (4) Mainstream social media | 0.427^**^ | 0.324^***^ | 0.446^***^ | 1.000 |
|  | (0.000) | (0.000) | (0.000) |  |
| (5) Alternative social media | 0.540^***^ | 0.447^***^ | 0.586^***^ | 0.646^***^ |
|  | (0.000) | (0.000) | (0.000) | (0.000) |

**** p<0.001*

**Table A9b. Point-biserial correlations with frequency of media use (by source)**

| Variables | (1) | (2) | (3) | (4) | (5) | (6) | (7) | (8) | (9) | (10) | (11) | (12) |
| --- | --- | --- | --- | --- | --- | --- | --- | --- | --- | --- | --- | --- |
| (1) Cryptocurrency | 1.000 |  |  |  |  |  |  |  |  |  |  |  |
|  |  |  |  |  |  |  |  |  |  |  |  |  |
| (2) Online newspapers | 0.276* | 1.000 |  |  |  |  |  |  |  |  |  |  |
|  | (0.000) |  |  |  |  |  |  |  |  |  |  |  |
| (3) Online news magazines | 0.368* | 0.645* | 1.000 |  |  |  |  |  |  |  |  |  |
|  | (0.000) | (0.000) |  |  |  |  |  |  |  |  |  |  |
| (4) Blogs | 0.447* | 0.450* | 0.562* | 1.000 |  |  |  |  |  |  |  |  |
|  | (0.000) | (0.000) | (0.000) |  |  |  |  |  |  |  |  |  |
| (5) YouTube | 0.344* | 0.206* | 0.347* | 0.434* | 1.000 |  |  |  |  |  |  |  |
|  | (0.000) | (0.000) | (0.000) | (0.000) |  |  |  |  |  |  |  |  |
| (6) Facebook | 0.191* | 0.173* | 0.268* | 0.346* | 0.517* | 1.000 |  |  |  |  |  |  |
|  | (0.000) | (0.000) | (0.000) | (0.000) | (0.000) |  |  |  |  |  |  |  |
| (7) Twitter | 0.446* | 0.399* | 0.479* | 0.548* | 0.473* | 0.352* | 1.000 |  |  |  |  |  |
|  | (0.000) | (0.000) | (0.000) | (0.000) | (0.000) | (0.000) |  |  |  |  |  |  |
| (8) Reddit | 0.469* | 0.400* | 0.516* | 0.604* | 0.437* | 0.289* | 0.581* | 1.000 |  |  |  |  |
|  | (0.000) | (0.000) | (0.000) | (0.000) | (0.000) | (0.000) | (0.000) |  |  |  |  |  |
| (9) Instagram | 0.370* | 0.286* | 0.407* | 0.499* | 0.562* | 0.471* | 0.613* | 0.521* | 1.000 |  |  |  |
|  | (0.000) | (0.000) | (0.000) | (0.000) | (0.000) | (0.000) | (0.000) | (0.000) |  |  |  |  |
| (10) TV news websites | 0.200* | 0.440* | 0.436* | 0.328* | 0.196* | 0.208* | 0.290* | 0.252* | 0.223* | 1.000 |  |  |
|  | (0.000) | (0.000) | (0.000) | (0.000) | (0.000) | (0.000) | (0.000) | (0.000) | (0.000) |  |  |  |
| (11) 8kun | 0.388* | 0.340* | 0.424* | 0.531* | 0.243* | 0.218* | 0.396* | 0.510* | 0.367* | 0.233* | 1.000 |  |
|  | (0.000) | (0.000) | (0.000) | (0.000) | (0.000) | (0.000) | (0.000) | (0.000) | (0.000) | (0.000) |  |  |
| (12) Telegram | 0.482* | 0.415* | 0.511* | 0.565* | 0.375* | 0.311* | 0.571* | 0.581* | 0.509* | 0.320* | 0.623* | 1.000 |
|  | (0.000) | (0.000) | (0.000) | (0.000) | (0.000) | (0.000) | (0.000) | (0.000) | (0.000) | (0.000) | (0.000) |  |
| (13) Truth Social | 0.401* | 0.354* | 0.455* | 0.562* | 0.327* | 0.288* | 0.465* | 0.522* | 0.444* | 0.295* | 0.678* | 0.657* |
|  | (0.000) | (0.000) | (0.000) | (0.000) | (0.000) | (0.000) | (0.000) | (0.000) | (0.000) | (0.000) | (0.000) | (0.000) |

** p<0.001*

**A11. Finding #10 – Logistic regression predicting cryptocurrency ownership**

**Table A10. Omnibus logistic regression predicting cryptocurrency ownership.**

| crypto | | Coef. | St.Err. | t-value | p-value | [95% Conf | | Interval] | | Sig |
| --- | --- | --- | --- | --- | --- | --- | --- | --- | --- | --- |
| Female | | -.753 | .14 | -5.40 | 0 | -1.027 | | -.48 | | *** |
| Age | | -.032 | .005 | -6.15 | 0 | -.042 | | -.022 | | *** |
| Income | | .118 | .042 | 2.78 | .005 | .035 | | .201 | | *** |
| Education | | .057 | .053 | 1.08 | .28 | -.046 | | .16 | |  |
| Religiosity | | .05 | .024 | 2.05 | .04 | .002 | | .098 | | ** |
| Patternicity | | .09 | .082 | 1.10 | .273 | -.07 | | .25 | |  |
| Desire for simple solutions | | .129 | .083 | 1.56 | .118 | -.033 | | .291 | |  |
| Confidence in science  community | | .051 | .078 | 0.65 | .515 | -.102 | | .204 | |  |
| Subjective numeracy | | .005 | .061 | 0.09 | .932 | -.115 | | .126 | |  |
| Total CTs believed | | .032 | .017 | 1.86 | .063 | -.002 | | .065 | | * |
| Anti-intellectualism | | .07 | .071 | 0.97 | .33 | -.07 | | .209 | |  |
| Argumentativeness | | .166 | .077 | 2.15 | .031 | .015 | | .317 | | ** |
| Narcissism | | .019 | .082 | 0.23 | .821 | -.143 | | .18 | |  |
| Need for Chaos | | .004 | .091 | 0.05 | .963 | -.174 | | .183 | |  |
| Schizotypal traits (cognitive) | | .026 | .091 | 0.28 | .778 | -.152 | | .203 | |  |
| Paranoia | | .055 | .079 | 0.70 | .484 | -.1 | | .211 | |  |
| Agentic goal pursuit | | .022 | .057 | 0.39 | .698 | -.089 | | .133 | |  |
| Conflict | | .052 | .048 | 1.09 | .276 | -.042 | | .146 | |  |
| Reactance | | -.018 | .085 | -0.21 | .833 | -.184 | | .149 | |  |
| Run for office | | -.022 | .065 | -0.35 | .729 | -.149 | | .104 | |  |
| Attend political meetings | | .101 | .121 | 0.84 | .403 | -.136 | | .337 | |  |
| Political volunteer | | .063 | .114 | 0.55 | .581 | -.16 | | .286 | |  |
| Gendered nationalism | | .039 | .075 | 0.52 | .602 | -.109 | | .187 | |  |
| Follows politics | | .087 | .071 | 1.24 | .215 | -.051 | | .226 | |  |
| Party ID (Dem-Rep) | | -.036 | .032 | -1.10 | .273 | -.099 | | .028 | |  |
| Contacted elected official | | -.056 | .095 | -0.59 | .556 | -.241 | | .13 | |  |
| Left-wing authoritarianism | | -.151 | .074 | -2.02 | .043 | -.296 | | -.005 | | ** |
| Right-wing authoritarianism | | -.239 | .086 | -2.77 | .006 | -.409 | | -.07 | | *** |
| Legacy media | | -.099 | .079 | -1.25 | .212 | -.254 | | .056 | |  |
| Online mainstream media | | .11 | .073 | 1.52 | .13 | -.032 | | .253 | |  |
| Mainstream social media | | .134 | .071 | 1.88 | .06 | -.005 | | .273 | | * |
| Alternative social media | | .489 | .102 | 4.78 | 0 | .289 | | .69 | | *** |
| Constant | | -2.701 | .771 | -3.50 | 0 | -4.213 | | -1.19 | | *** |
|  | | | | | | | | | | |
| Mean dependent var | 0.298 | | | SD dependent var | | | 0.458 | |  |  |
| Pseudo r-squared | 0.324 | | | Number of obs | | | 1982 | |  |  |
| Chi-square | 782.961 | | | Prob > chi2 | | | 0.000 | |  |  |
| Akaike crit. (AIC) | 1698.373 | | | Bayesian crit. (BIC) | | | 1882.904 | |  |  |
| **** p<.01, ** p<.05, * p<.1* | | | | | | | | | | |
|  | | | | | | | | | | |

**A12. Logistic regression models predicting cryptocurrency ownership from each group of variables by type (conspiracism and thinking styles, political variables, and personality/motivational variables)**

**
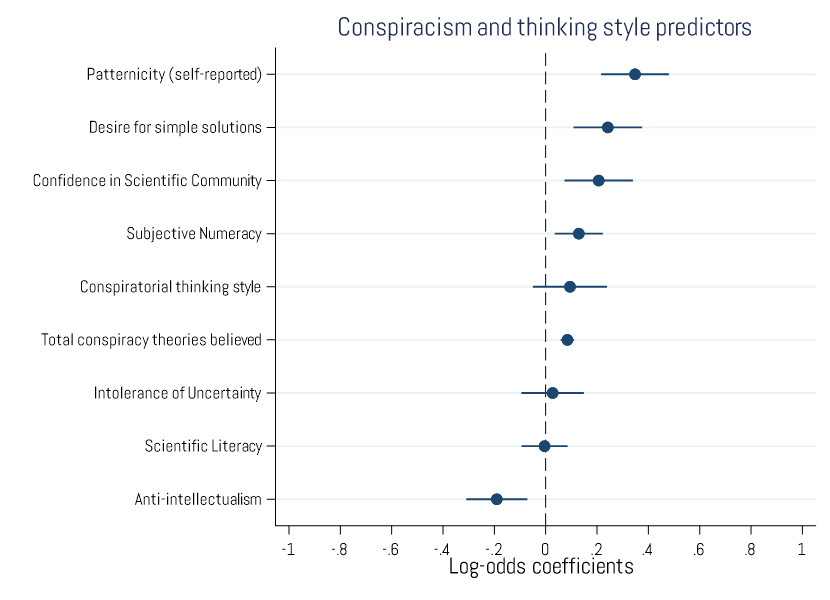
**

**Figure A1. Logistic regression predicting cryptocurrency ownership from conspiracism and thinking styles**. Coefficients are log odds. Error bars represent 95% confidence intervals. N=2,001.

**Figure A2. Logistic regression predicting cryptocurrency ownership from political variables (excluding feeling thermometer attitudes)**. Coefficients are log odds. Error bars represent 95% confidence intervals. N=2,001.

**Figure A3. Logistic regression predicting cryptocurrency ownership from personality and motivational variables**. Coefficients are log odds. Error bars represent 95% confidence intervals. N=2,001.

**A13. Correlations between cryptocurrency ownership and belief in specific conspiracy theories; also total conspiracy theories believed by participants**


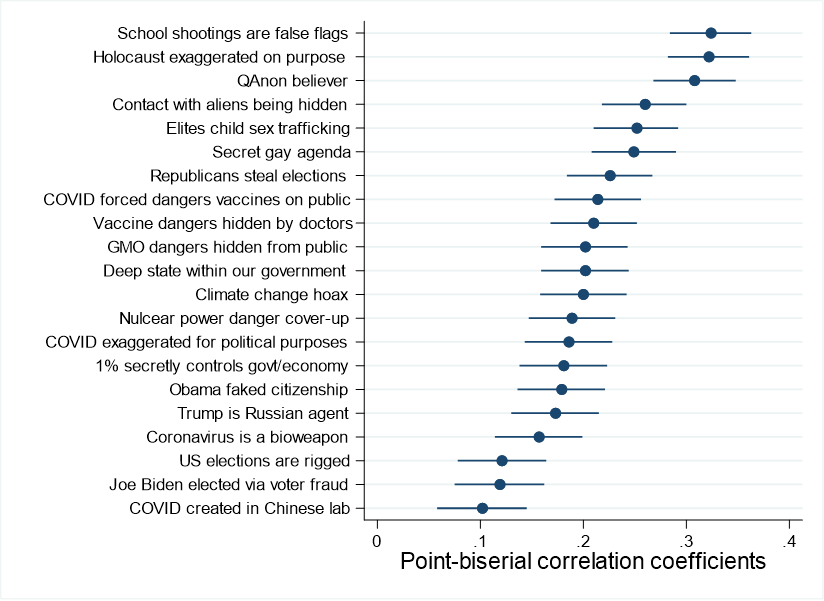


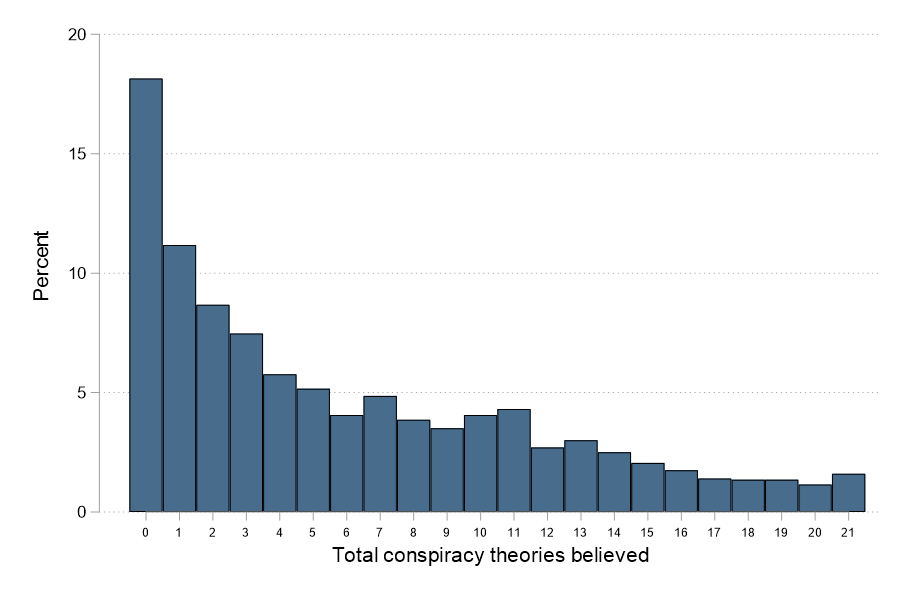


**References**

1. Petersen, M.B., Osmundsen, M., & Arceneaux, K. (2020). The ‘need for chaos’ and motivations to share hostile political rumors. *PsyArxiv,* <https://psyarxiv.com/6m4ts/> [↑](#endnote-ref-1)
2. Jonason, P. K., & Webster, G.D. (2010). The dirty dozen: A concise measure of the dark triad. *Psychological Assessment 22*(2), 420-432. <https://doi.org/10.1037/a0019265> [↑](#endnote-ref-2)
3. Paulhus, D. L., Buckels, E. E., Trapnell, P. D., & Jones, D. N. (2021). Screening for dark personalities: The Short Dark Tetrad (SD4). *European Journal of Psychological Assessment, 37*(3), 208–222. <https://doi.org/10.1027/1015-5759/a000602> [↑](#endnote-ref-3)
4. Green, C. E., Freeman, D., Kuipers, E., Bebbington, P., Fowler, D., Dunn, G., & Garety, P. A. (2008). Measuring ideas of persecution and social reference: the Green et al. Paranoid Thought Scales (GPTS). *Psychological Medicine, 38*(1), 101–111. <https://doi.org/10.1017/S0033291707001638> [↑](#endnote-ref-4)
5. McClosky, H., & Chong, D. (1985). Similarities and differences between left-wing and right-wing radicals. *British Journal of Political Science, 15*(3), 329-363. <https://doi.org.10.1017/S0007123400004221> [↑](#endnote-ref-5)
6. Edelson, J., Alduncin, A., Krewson, C., Sieja, J. A., & Uscinski, J. E. (2017). The effect of conspiratorial thinking and motivated reasoning on belief in election fraud. *Political Research Quarterly, 70*(4), 933–946. <https://doi.org/10.1177/1065912917721061> [↑](#endnote-ref-6)
7. Bizumic, B., & Duckitt, J. (2018). Investigating Right Wing Authoritarianism with a very short authoritarianism scale. *Journal of Social and Political Psychology, 6*(1), 129-150. <https://doi.org/10.5964/jspp.v6i1.835> [↑](#endnote-ref-7)
8. Costello, T. H., Bowes, S. M., Stevens, S. T., Waldman, I. D., Tasimi, A., & Lilienfeld, S. O. (2022). Clarifying the structure and nature of left-wing authoritarianism. *Journal of Personality and Social Psychology, 122*(1), 135–170. <https://doi.org/10.1037/pspp0000341> [↑](#endnote-ref-8)
9. Armaly, M. T., & Enders, A. M. (2022). 'Why Me?' The role of perceived victimhood in American politics. *Political Behavior, 44*(4), 1583–1609. <https://doi.org/10.1007/s11109-020-09662-x> [↑](#endnote-ref-9)
10. Conrad, K. J., Riley, B. B., Conrad, K. M., Chan, Y. F., & Dennis, M. L. (2010). Validation of the Crime and Violence Scale (CVS) against the Rasch measurement model including differences by gender, race, and age. *Evaluation Review, 34*(2), 83–115. <https://doi.org/10.1177/0193841X10362162> [↑](#endnote-ref-10)
11. Uscinski, J. E., Enders, A. M., Seelig, M. I., Klofstad, C. A., Funchion, J. R., Everett, C., ... & Murthi, M. N. (2021). American politics in two dimensions: Partisan and ideological identities versus anti‐establishment orientations. *American Journal of Political Science, 65*(4), 877-895. <https://doi.org/10.1111/ajps.12616> [↑](#endnote-ref-11)
12. Buhr, K., & Dugas, M. J. (2002). The intolerance of uncertainty scale: Psychometric properties of the English version. *Behaviour Research and Therapy, 40*(8), 931-945. <https://doi.org/10.1016/S0005-7967(01)00092-4> [↑](#endnote-ref-12)
13. Durand, M. A., Yen, R. W., O’Malley, J., Elwyn, G., & Mancini, J. (2020). Graph literacy matters: Examining the association between graph literacy, health literacy, and numeracy in a Medicaid eligible population. *PloS One, 15*(11), e0241844. <https://doi.org/10.1371/journal.pone.0241844> [↑](#endnote-ref-13)
14. Chun, J. W., & Lee, M. J. (2017). When does individuals’ willingness to speak out increase on social media? Perceived social support and perceived power/control. *Computers in Human Behavior, 74*, 120-129. <https://doi.org/10.1016/j.chb.2017.04.010> [↑](#endnote-ref-14)
15. Choi, T. R., & Sung, Y. (2018). Instagram versus Snapchat: Self-expression and privacy concern on social media. *Telematics and Informatics, 35*(8), 2289–2298. <https://doi.org/10.1016/j.tele.2018.09.009> [↑](#endnote-ref-15)
16. Crawford, J. R., & Henry, J. D. (2004). The Positive and Negative Affect Schedule (PANAS): Construct validity, measurement properties and normative data in a large non‐clinical sample. *British Journal of Clinical Psychology, 43*(3), 245-265. <https://doi.org/10.1348/0144665031752934> [↑](#endnote-ref-16)
17. Okamoto, S., Niwa, F., Shimizu, K., & Sugiman, T. (2001). *The 2001 Survey for Public Attitudes Towards and Understanding of Science and Technology in Japan.* NISTEP Report 72. [↑](#endnote-ref-17)
